# Supplementary material for: Influence of Electronic Environment on the Radiative Efficiency of 9-Phenyl-9H-carbazole-Based ortho-Carboranyl Luminophores
Source: Molecules. 2021 Mar 21;26(6):1763. doi: 10.3390/molecules26061763 (PMC8003977; doi:10.3390/molecules26061763)
Supplement: Supplementary file 1 [file molecules-26-01763-s001.pdf]

Supplementary Material

Influence of Electronic Environment on the Radiative Efficiency of 9-Phenyl-9*H*-carbazole-Based *ortho*-Carboranyl Luminophores

Seok Ho Lee,[1,†] Ji Hye Lee,[1,†] Min Sik Mun,[1,†] Sanghee Yi,[1] Eunji Yoo,[1] Hyonseok Hwang,[1] and Kang Mun Lee[1,*]

[1] Department of Chemistry, Institute for Molecular Science and Fusion Technology, Kangwon National University, Chuncheon 24341, Republic of Korea.

*****Correspondence: kangmunlee@kangwon.ac.kr (K.M.L.); Tel.: +82-33-250-8499 (K.M.L)
†The first, second and third authors contributed equally to this work.

Contents

Multinuclear NMR spectra for *o*-carboranyl compounds and their precursors ··············· S2–S15
Crystallographic data and parameters for **1F** and **4T** ··············· S16
Selected bond lengths (Å) and angles (°) for **1F** and **4T** ··············· S17
UV-vis absorption and PL spectra for 9-phenyl-9*H*-carbazole ··············· S18
Emission decay curves for *o*-carboranyl compounds in film state ··············· S19
Theoretical calculation results for *o*-carboranyl compounds ··············· S20–S31
Cartesian coordinates for *o*-carboranyl compounds ··············· S32–S39

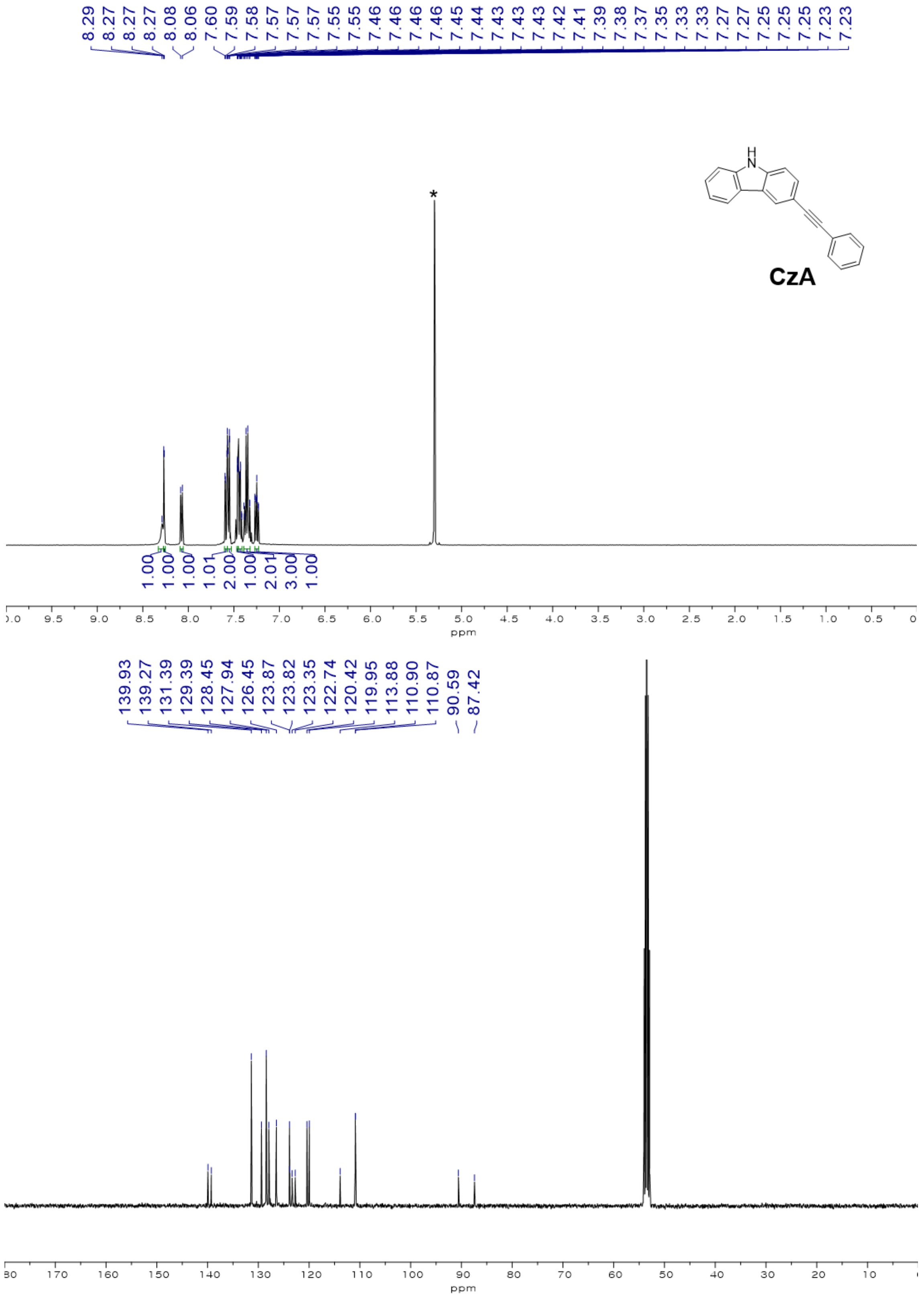

Figure S1. ^{1}H (top) and ^{13}C (bottom) NMR spectra of **CzA** in CD_2Cl_2 (* from residual CH_2Cl_2 in CD_2Cl_2).

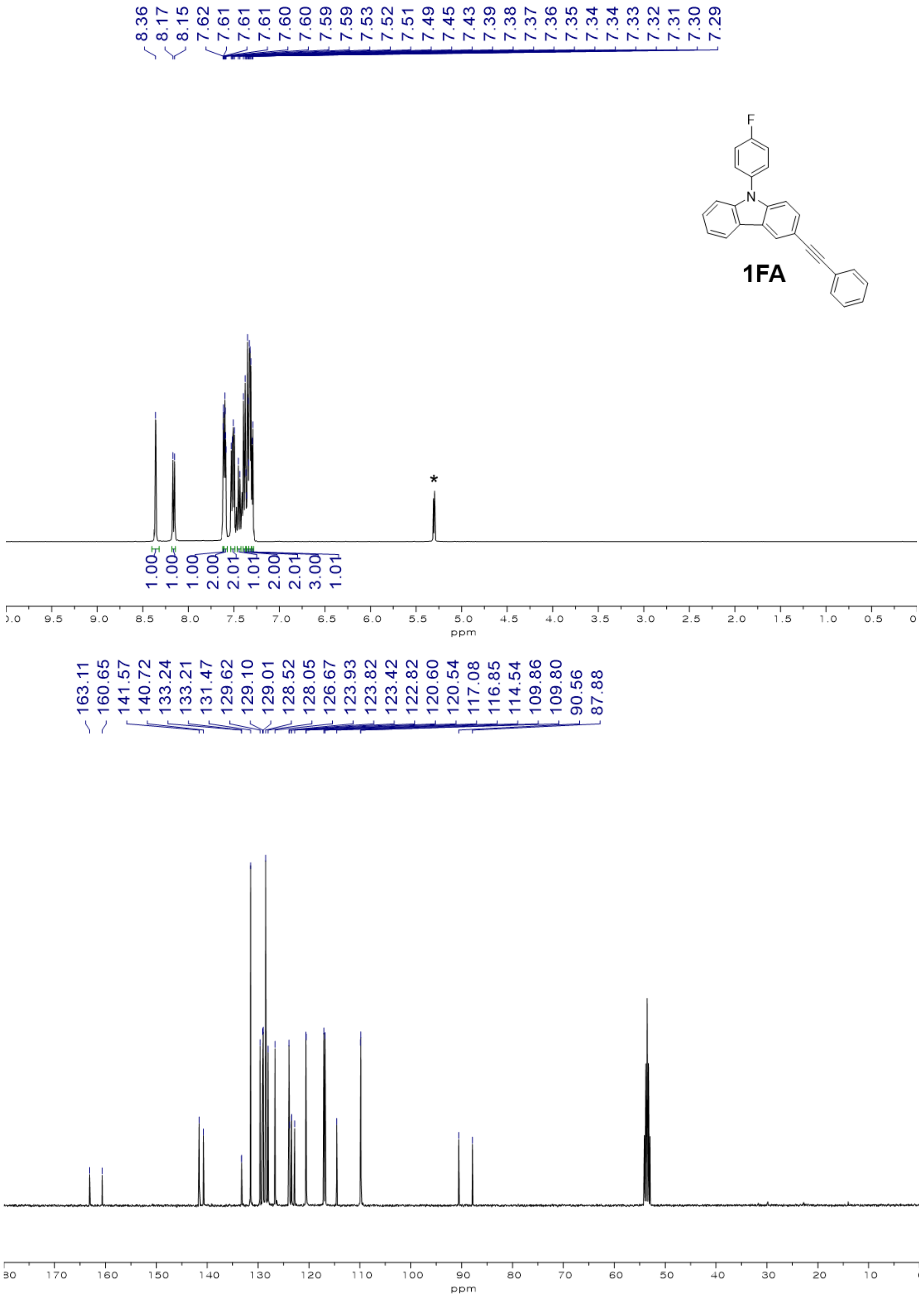

Figure S2. ^{1}H (top) and ^{13}C (bottom) NMR spectra of **1FA** in CD_2Cl_2 (* from residual CH_2Cl_2 in CD_2Cl_2).

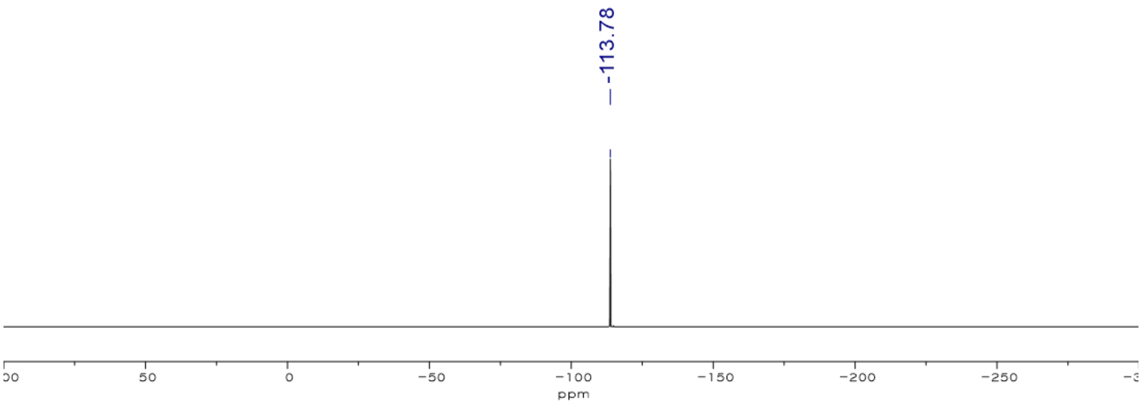

Figure S3. ^{19}F NMR spectra of **1FA** in CD_2Cl_2.

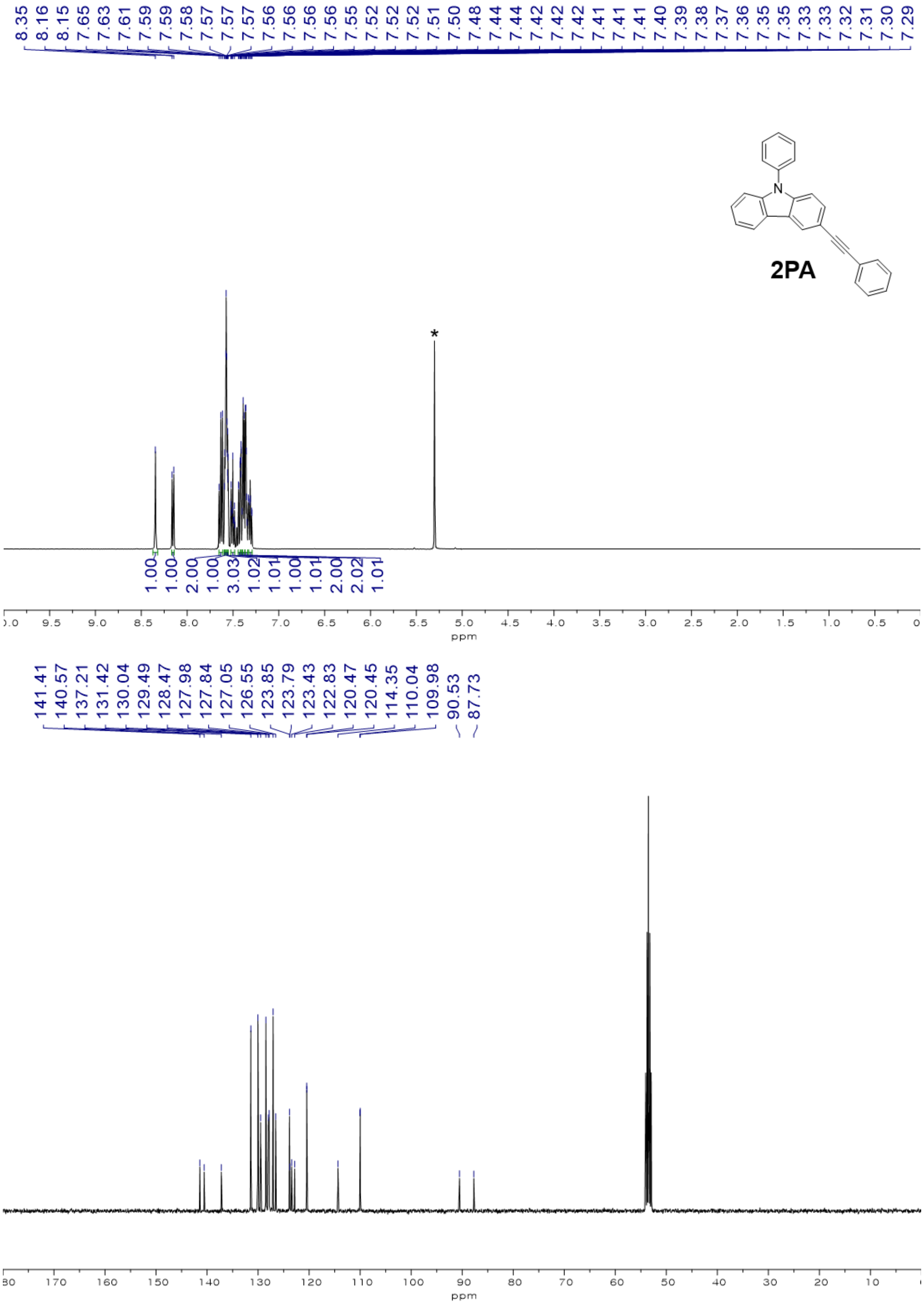

Figure S4. ^{1}H (top) and ^{13}C (bottom) NMR spectra of **2PA** in CD_2Cl_2 (* from residual CH_2Cl_2 in CD_2Cl_2).

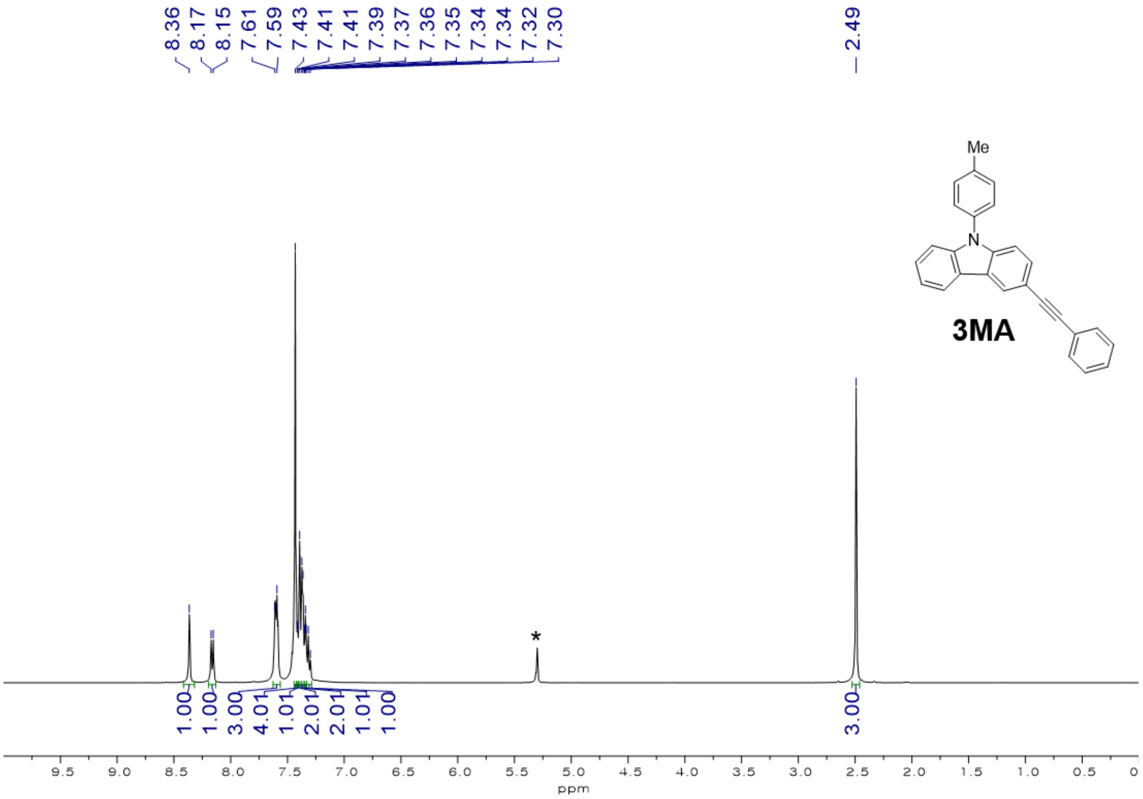

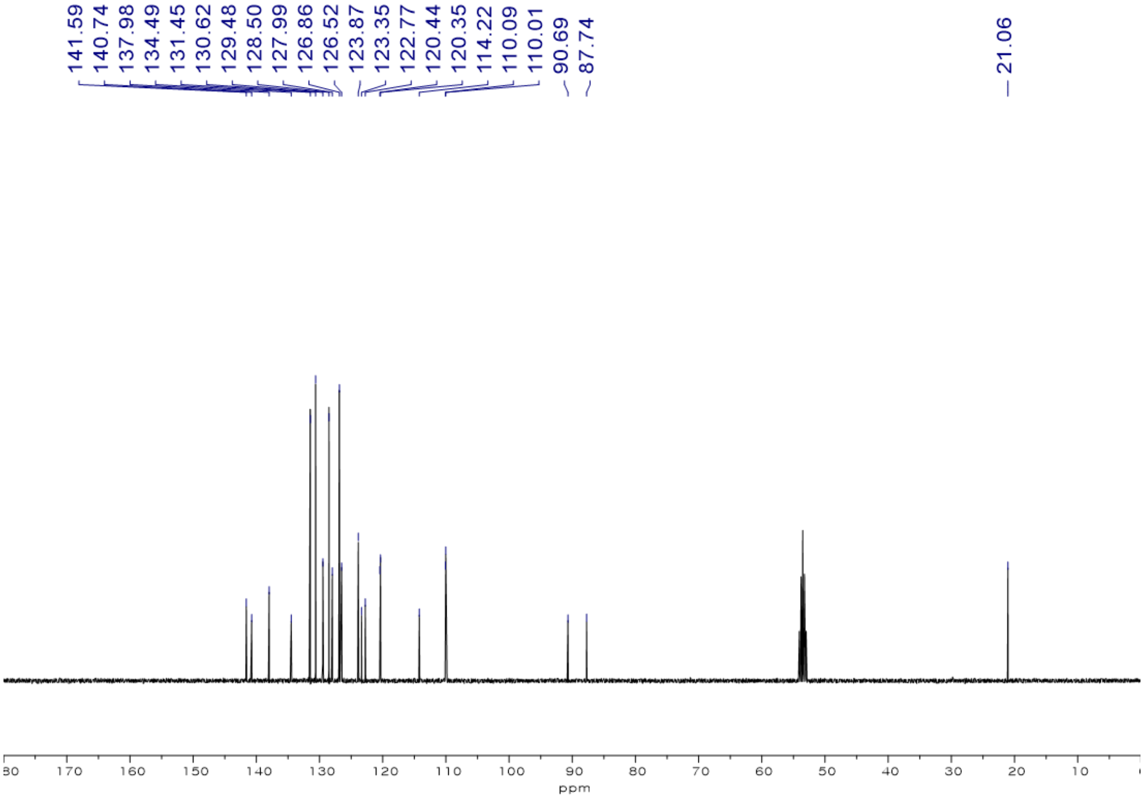

Figure S5. ^{1}H (top) and ^{13}C (bottom) NMR spectra of **3MA** in CD_2Cl_2 (* from residual CH_2Cl_2 in CD_2Cl_2).

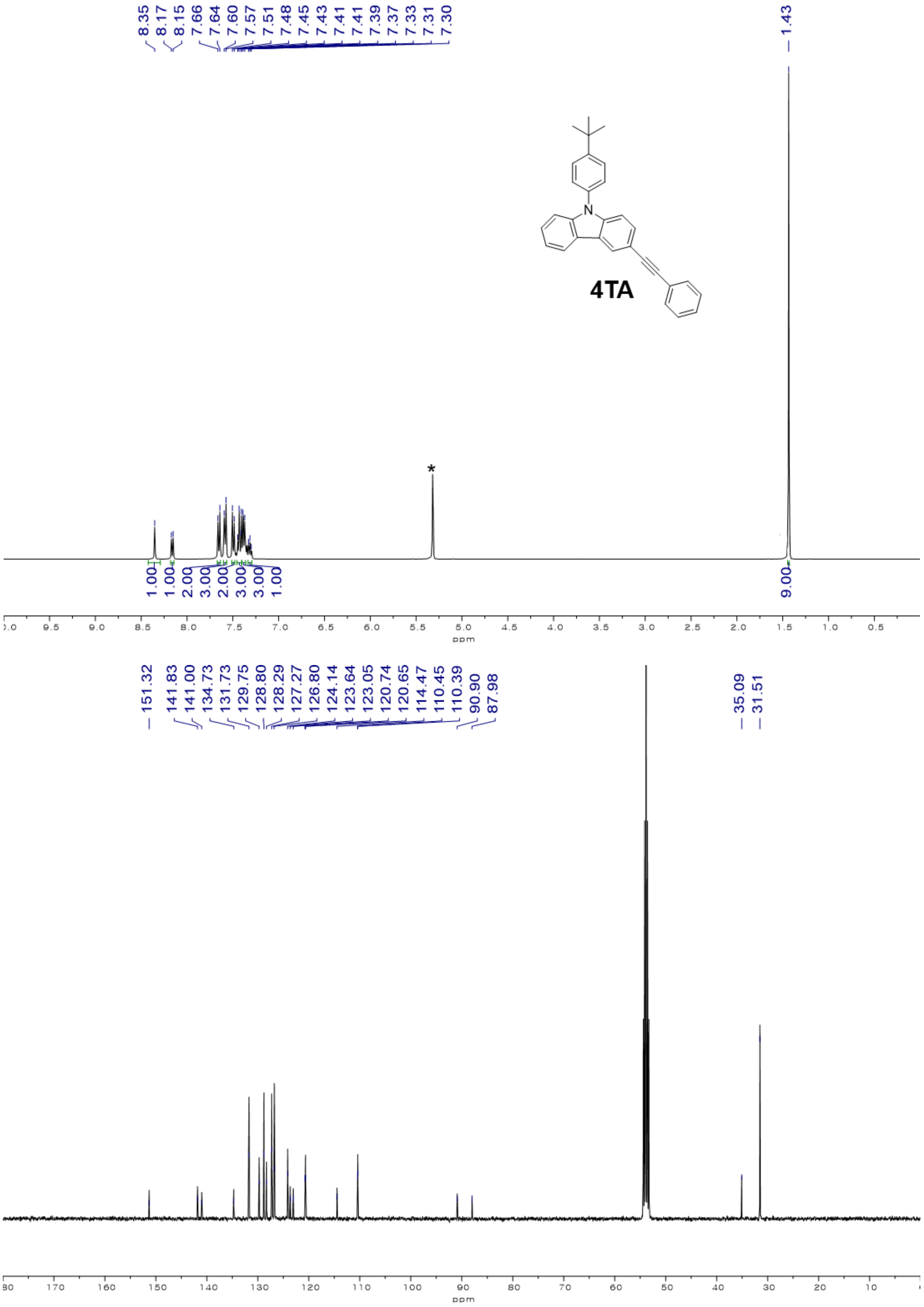

Figure S6. ^{1}H (top) and ^{13}C (bottom) NMR spectra of **4TA** in CD_2Cl_2 (* from residual CH_2Cl_2 in CD_2Cl_2).

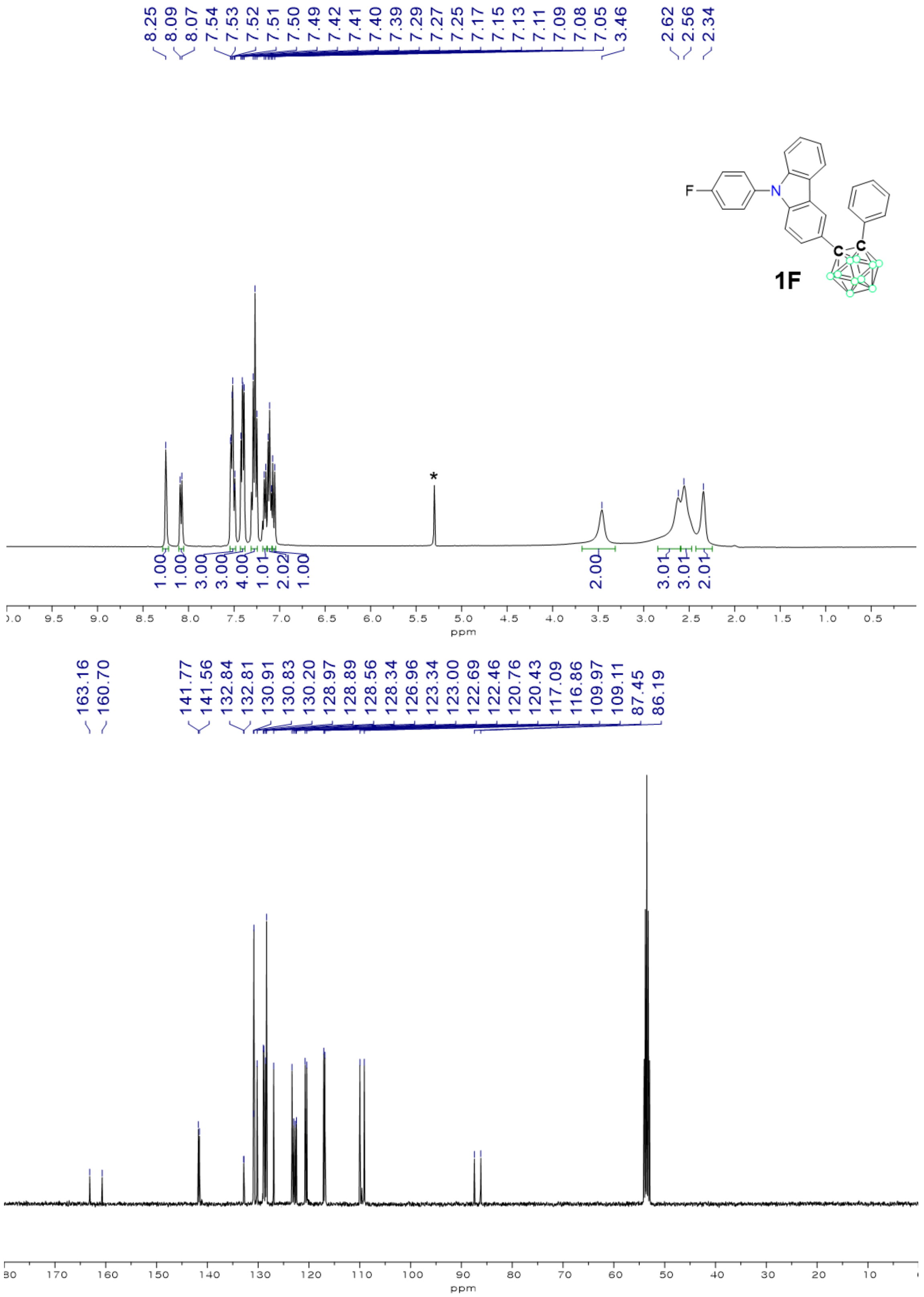

Figure S7. $^{1}H\{^{11}B\}$ (top) and ^{13}C (bottom) NMR spectra of **1F** in CD_2Cl_2 (* from residual CH_2Cl_2 in CD_2Cl_2).

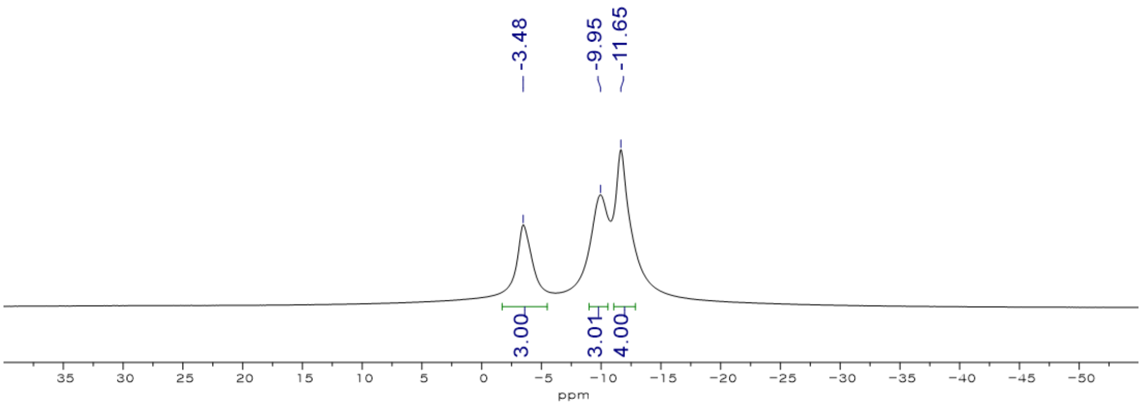

Figure S8. $^{11}B\{^{1}H\}$ NMR spectra of **1F** in CD_2Cl_2.

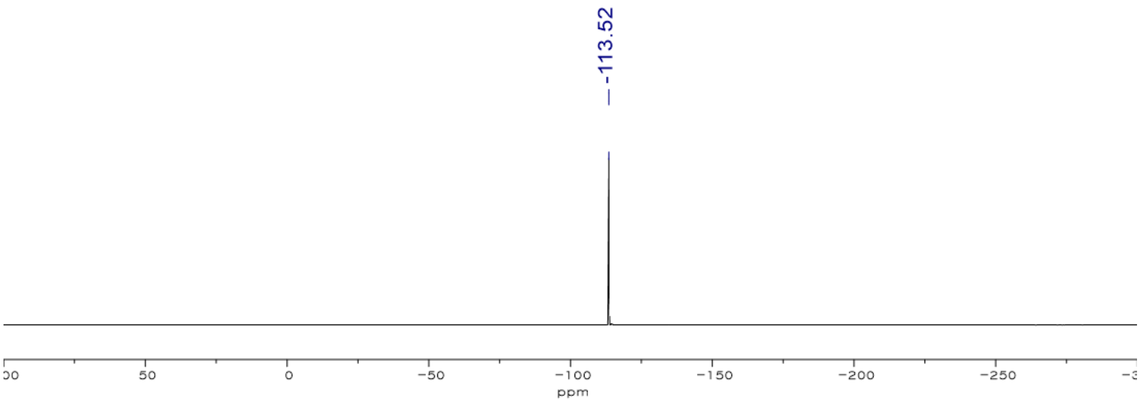

Figure S9. ^{19}F NMR spectra of **1F** in CD_2Cl_2.

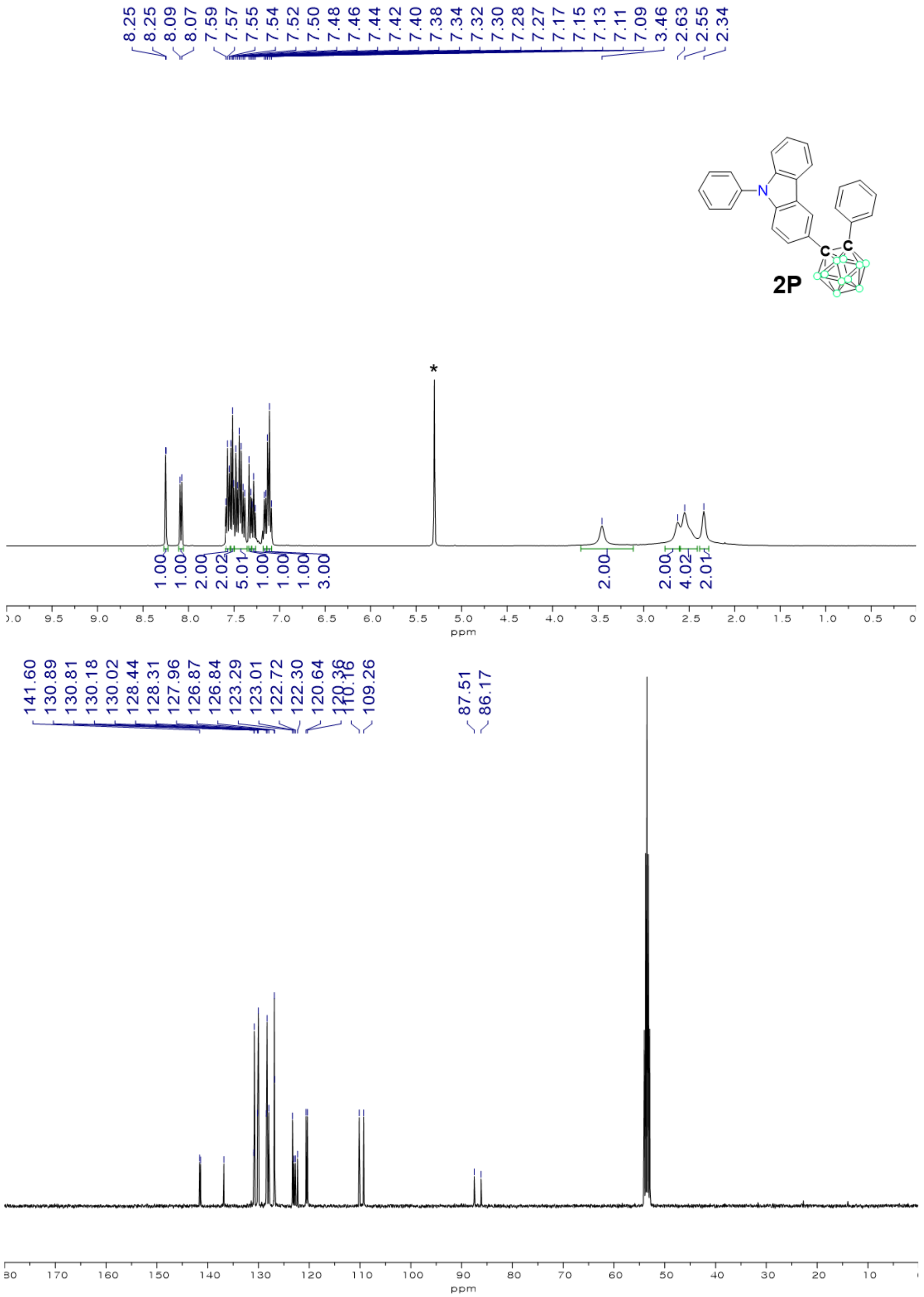

Figure S10. ^{1}H{^{11}B} (top) and ^{13}C (bottom) NMR spectra of **2P** in CD_2Cl_2 (* from residual CH_2Cl_2 in CD_2Cl_2).

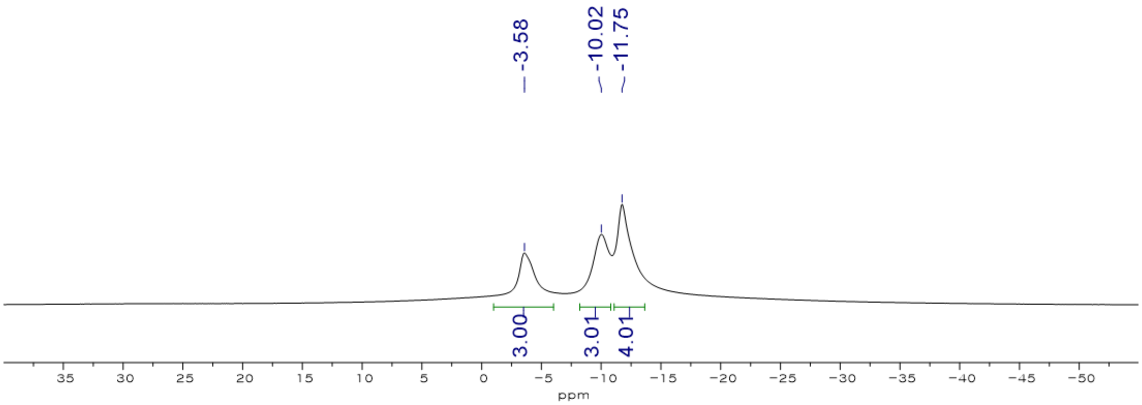

Figure S11. $^{11}B\{^{1}H\}$ NMR spectra of **2P** in CD_2Cl_2.

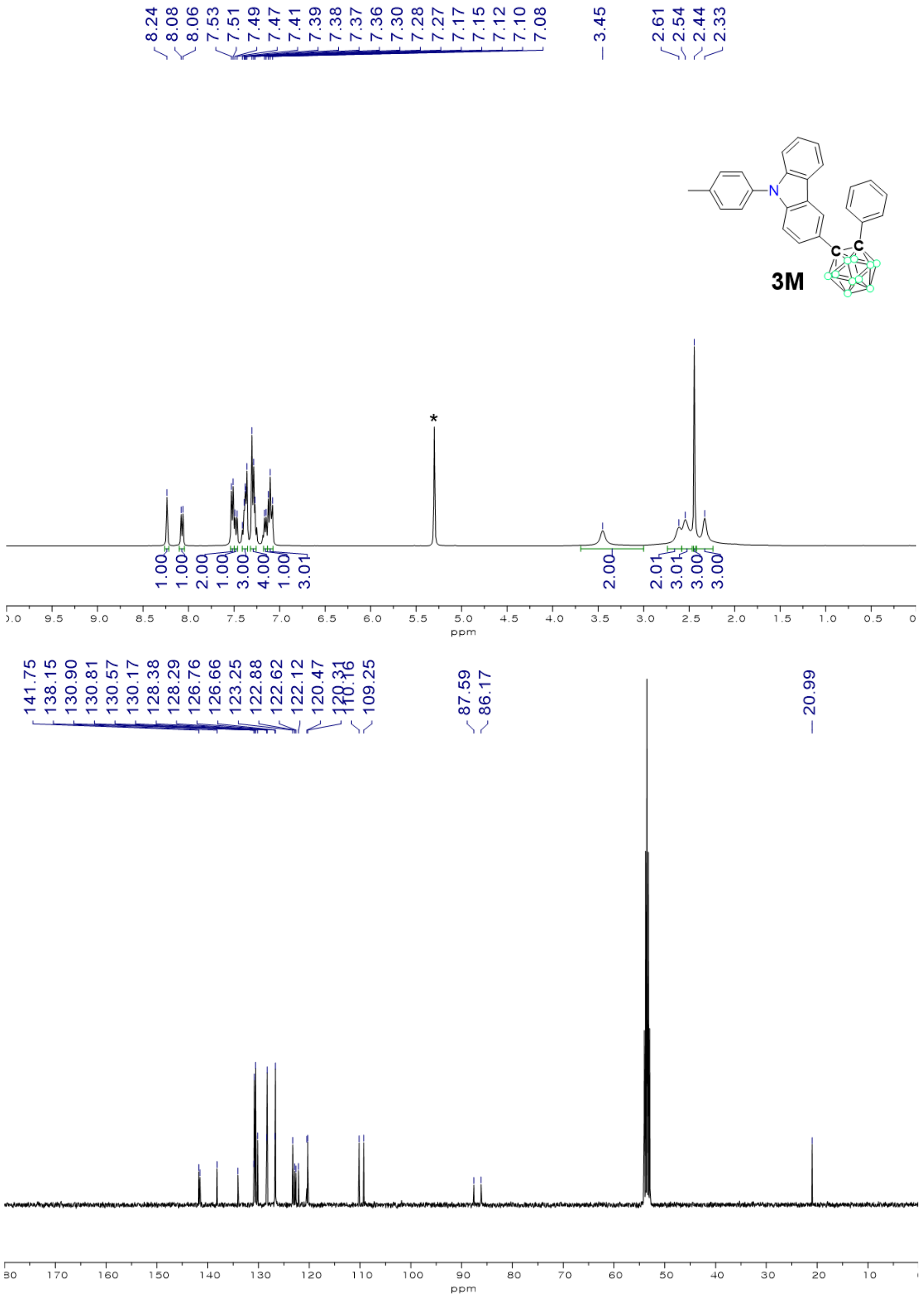

Figure S12. $^{1}H\{^{11}B\}$ (top) and ^{13}C (bottom) NMR spectra of **3M** in CD_2Cl_2 (* from residual CH_2Cl_2 in CD_2Cl_2).

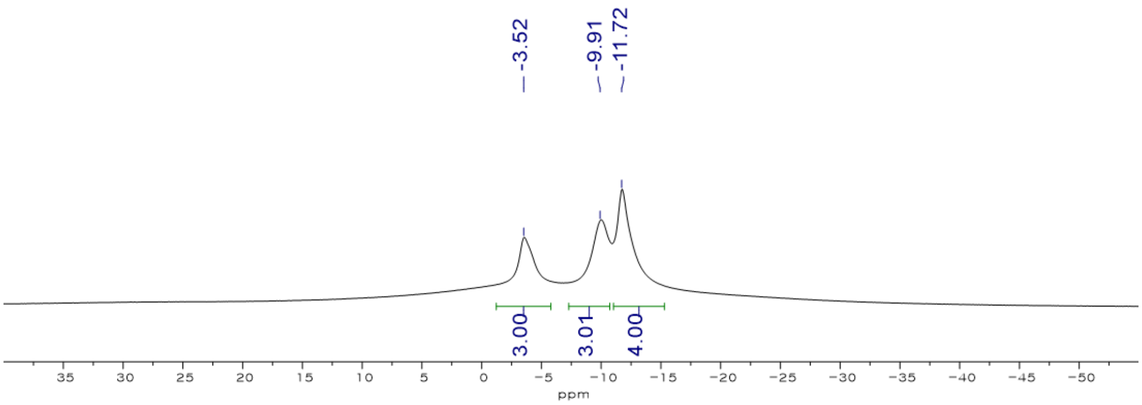

Figure S13. $^{11}B\{^{1}H\}$ NMR spectra of **3M** in CD_2Cl_2.

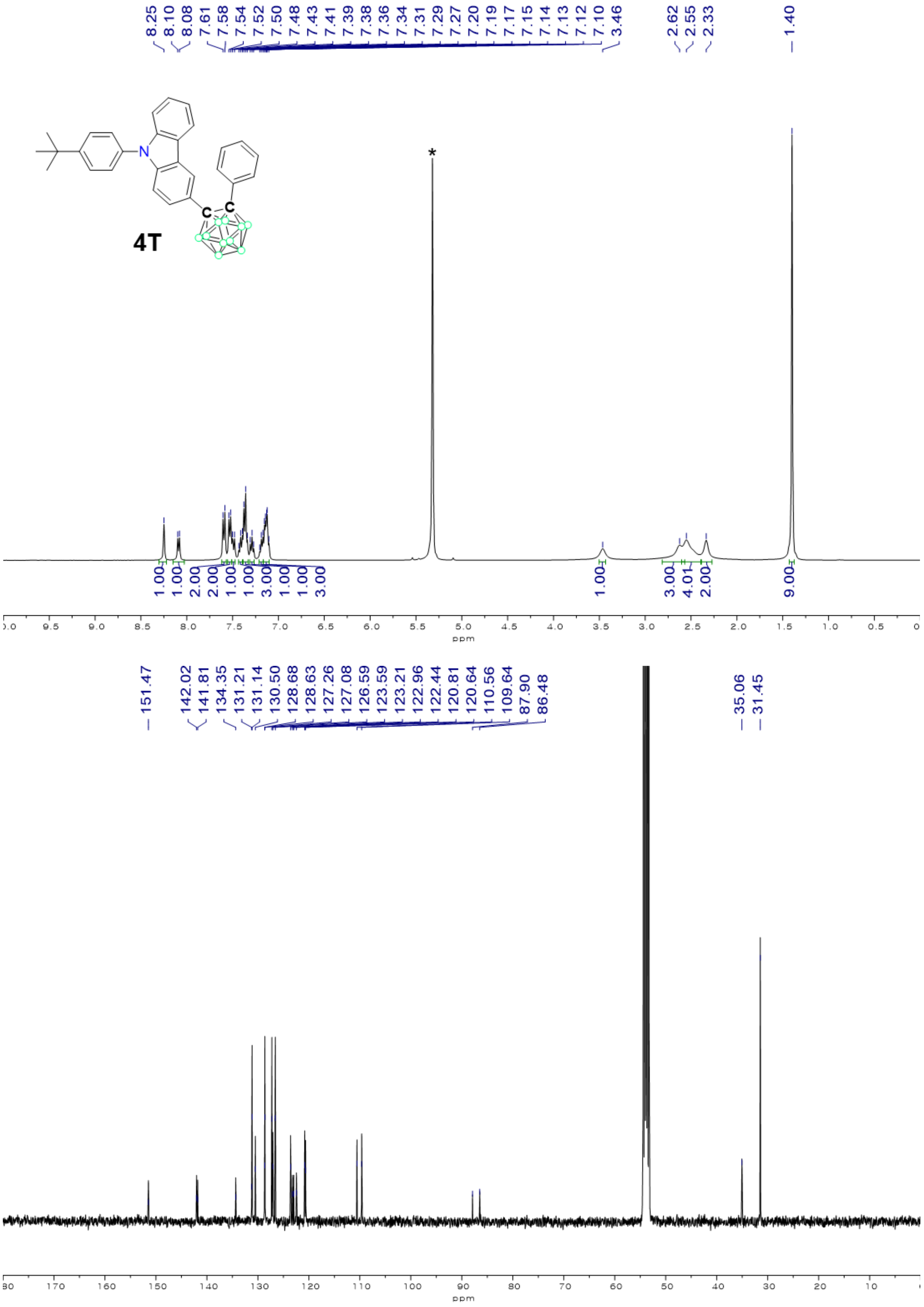

Figure S14. $^{1}H\{^{11}B\}$ (top) and ^{13}C (bottom) NMR spectra of **4T** in CD_2Cl_2 (* from residual CH_2Cl_2 in CD_2Cl_2).

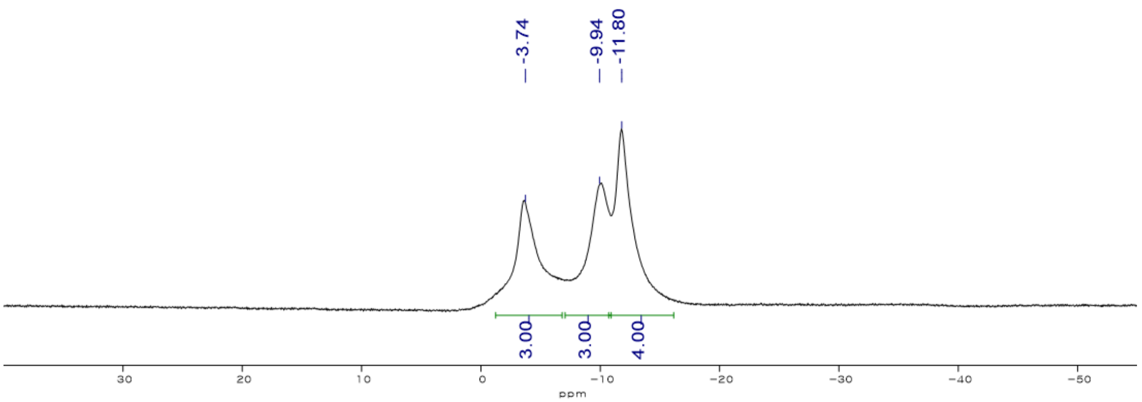

Figure S15. $^{11}B\{^1H\}$ NMR spectra of **4T** in CD_2Cl_2.

Table S1. Crystallographic data and parameters for **1F** and **4T**

Compound	**1F**	**4T**
Formula	$C_{26}H_{26}B_{10}FN$	$C_{30}H_{35}B_{10}N$
Formula weight	479.58	517.69
Crystal system	Triclinic	Triclinic
Space group	P_{-1}	P_{-1}
a (Å)	7.9390(16)	10.106(2)
b (Å)	12.778(3)	12.302(2)
c (Å)	14.444(3)	13.052(3)
α (°)	107.95(3)	91.32(3)
β (°)	98.07(3)	107.27(3)
γ (°)	106.74(3)	105.49(3)
V (Å^3)	1292.0(5)	1484.0(6)
Z	2	2
ρ_{calc}(g cm^{-3})	1.233	1.159
μ (mm^{-1})	0.070	0.061
F(000)	496	544
T (K)	293(2)	293(2)
Scan mode	*phi and omega scans*	*phi and omega scans*
hkl range	$-10 < h < 10$, $-16 < k < 16$, $-18 < l < 18$	$-12 < h < 13$, $-15 < k < 15$, $-16 < l < 16$
Measd reflns	13002	14919
Unique reflns [R_{int}]	5904 [0.0439]	6786 [0.1185]
Reflns used for refinement	5904	6786
Refined parameters	343	404
$R_1{}^1$ (I > 2σ(I))	0.0556	0.0695
$wR_2{}^2$ all data	0.1586	0.2033
GOF on F^2	1.004	1.001
ρ_{fin} (max/min) (e Å^{-3})	0.273, −0.190	0.236, −0.367

$^1R_1 = \sum||Fo| - |Fc||/\sum|Fo|$. $^2wR_2 = \{[\sum w(Fo^2 - Fc^2)^2]/[\sum w(Fo^2)^2]\}^{1/2}$.

Table S2. Selected bond lengths (Å) and angles (°) for **1F** and **4T**

Compound	**1F**	**4T**
length (Å)		
C3–C13	1.5043(19)	1.500(3)
C13–C14	1.7331(19)	1.740(3)
C14–C15	1.501(2)	1.507(3)
angles (°)		
C3–C13–C14	117.70(11)	122.36(19)
C13–C14–C15	117.46(11)	119.2(2)
C9–N1–C12	107.98(13)	108.1(2)
C9–N1–C21	126.34(13)	126.3(2)
C12–N1–C21	124.82(13)	125.1(2)

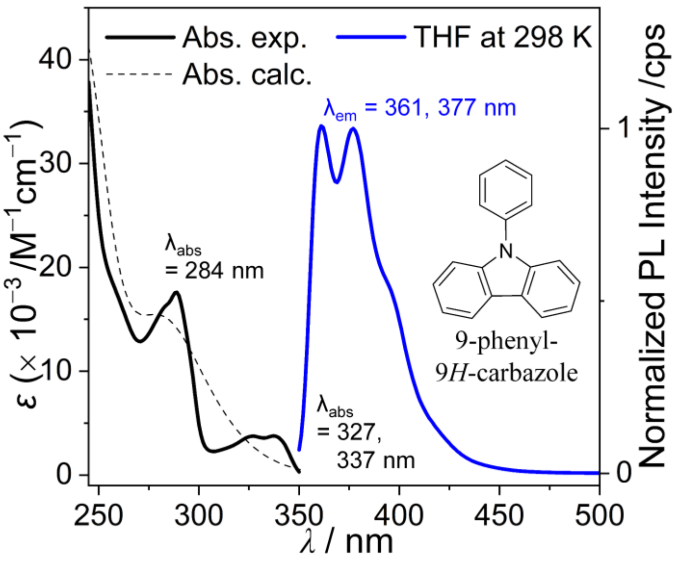

Figure S16. UV-vis absorption (left side) and PL spectra (right side) for 9-phenyl-9*H*-carbazole (λ_{ex} = 327 nm). Black line: absorption spectra in THF (3.0 × 10^{-5} M), black dash-line: absorption spectra calculated using TD-DFT, and blue line: PL spectra in THF (3.0 × 10^{-5} M) at 298 K.

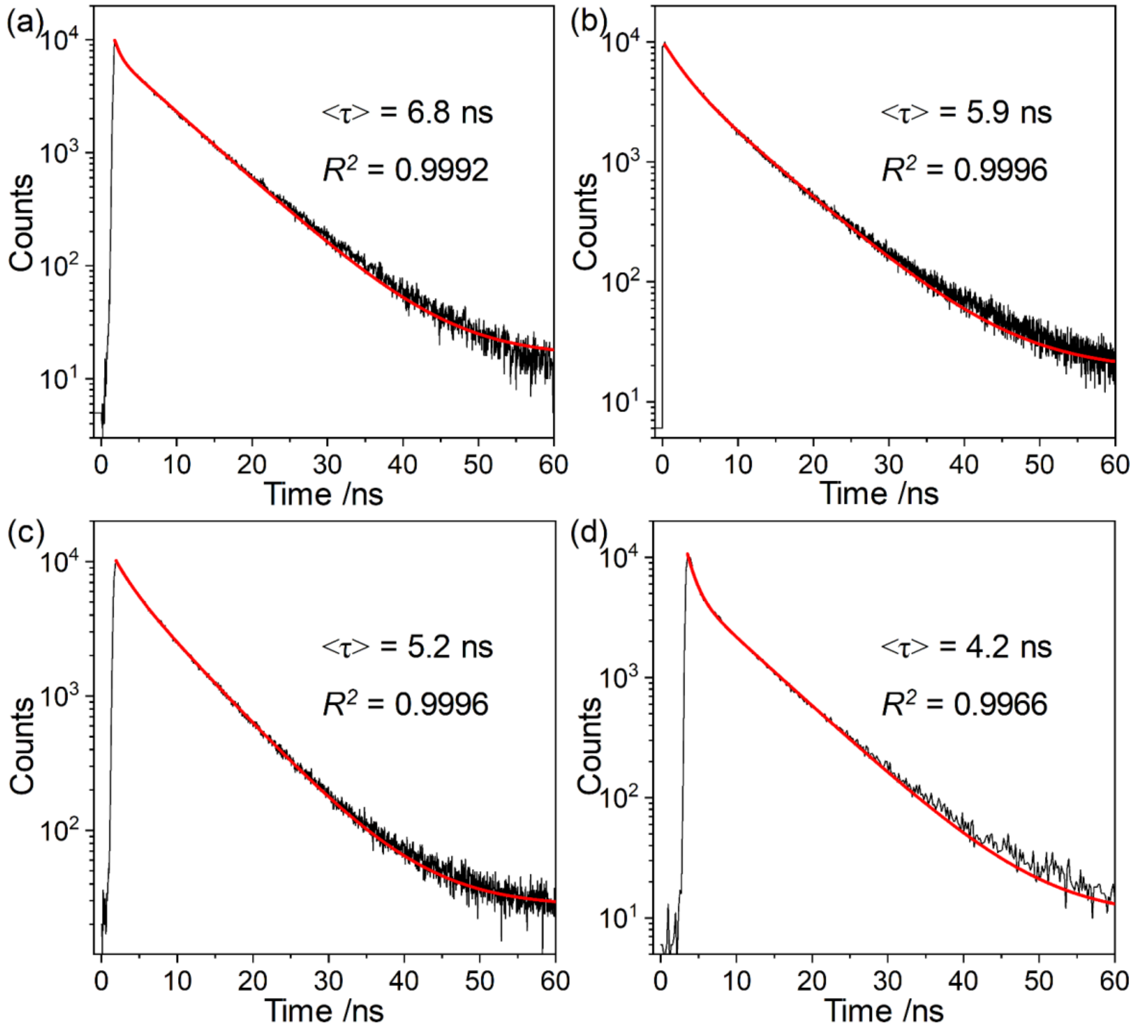

Figure S17. Emission decay curves for (a) **1F**, (b) **2P**, (c) **3M**, and (d) **4T** in the film state (5 wt% doped in PMMA) detected at each CT based emission maxima at 298 K. Each red-line is its single exponential fitting curve for the decay curves.

Computational calculation details

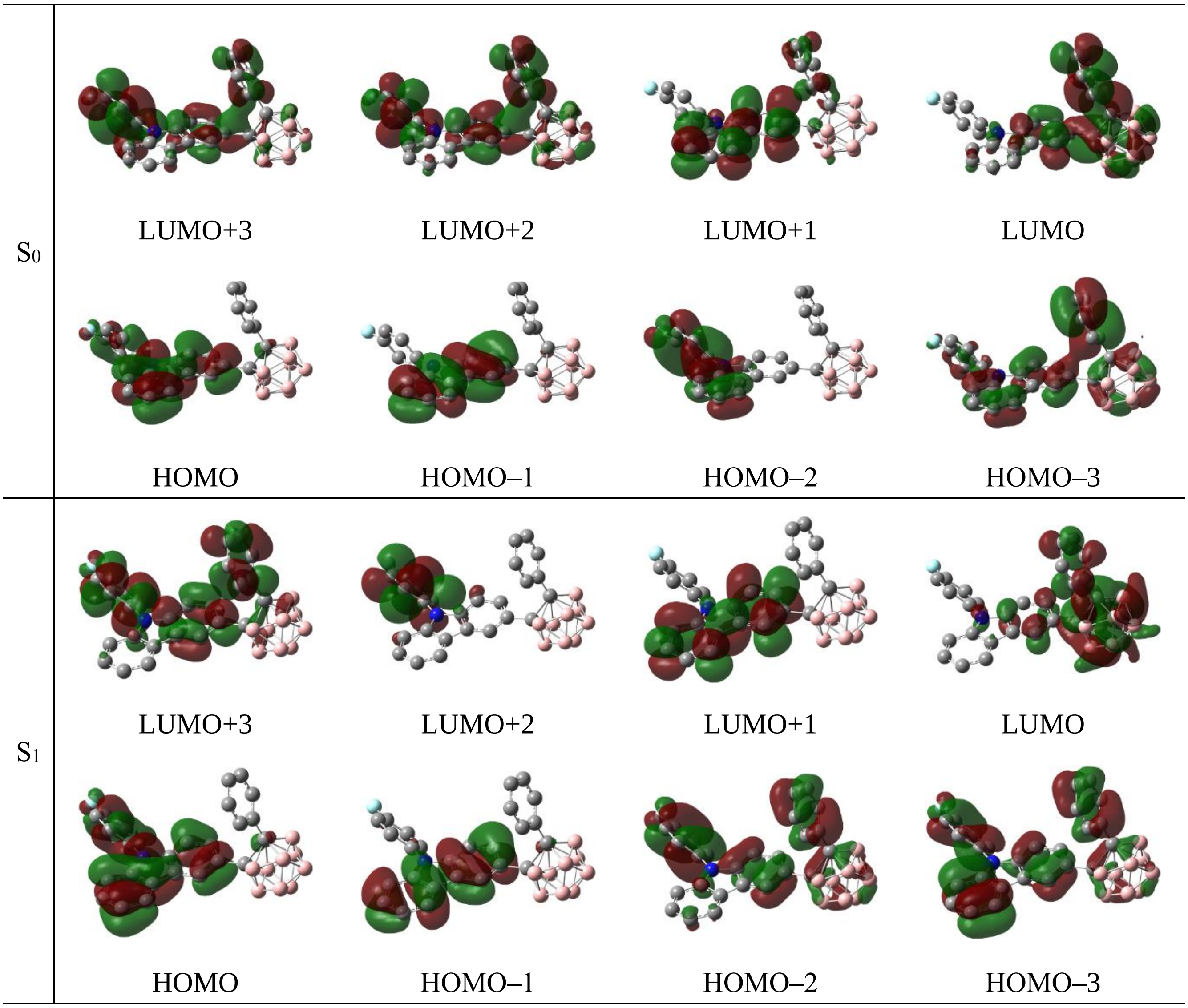

Figure S18. The selected frontier orbitals of **1F** from B3LYP calculations (Isovalue = 0.04 a.u.) at the ground state (S_0) and first singlet excited state (S_1) optimized geometries in THF.

Table S3. Computed absorption wavelengths (λ_{calc} in nm) and oscillator strengths ($f_{calc.}$) for **1F** from TD-B3LYP calculations using the B3LYP geometries at the ground state (S_0) and first singlet excited state (S_1) optimized geometries in THF

state	λ_{calc} (/nm)	f_{calc}	Major contribution		
			S_0		
1	320.85	0.1359	HOMO	→	LUMO (84.7%)
			HOMO	→	LUMO+1 (10.7%)
2	312.76	0.0805	HOMO−1	→	LUMO (9.1%)
			HOMO	→	LUMO (11.5%)
			HOMO	→	LUMO+1 (73.8%)
3	289.51	0.0953	HOMO−1	→	LUMO (79.0%)
			HOMO	→	LUMO+1 (9.0%)
4	281.45	0.0081	HOMO	→	LUMO+2 (56.8%)
			HOMO	→	LUMO+3 (33.7%)
5	278.67	0.0664	HOMO−1	→	LUMO (6.8%)
			HOMO−1	→	LUMO+1 (27.0%)
			HOMO	→	LUMO+2 (10.8%)
			HOMO	→	LUMO+3 (50.4%)
			S_1		
1	557.58	0.2016	HOMO	→	LUMO (98.8%)
2	413.59	0.1582	HOMO−1	→	LUMO (98.3%)
3	388.30	0.0534	HOMO−3	→	LUMO (49.1%)
			HOMO−2	→	LUMO (47.0%)
4	382.30	0.0052	HOMO−3	→	LUMO (96.6%)
5	374.76	0.0108	HOMO−4	→	LUMO (77.7%)
			HOMO−2	→	LUMO (21.3%)

Table S4. Molecular orbital energies (in eV) and molecular orbital distributions (in %) of **1F** at the ground state (S_0) and first singlet excited state (S_1) optimized geometries in THF

	E (eV)	*o*-carborane	phenyl carbazole	carborane phenyl
		S_0		
LUMO+3	−0.66	5.6	83.8	10.6
LUMO+2	−0.69	9.1	72.8	18.1
LUMO+1	−1.14	5.0	88.8	6.2
LUMO	−1.36	34.2	41.3	24.6
HOMO	−5.76	2.6	97.3	0.1
HOMO−1	−6.16	1.2	98.5	0.2
HOMO−2	−7.08	0.0	100.0	0.0
HOMO−3	−7.12	9.6	30.0	60.4
		S_1		
LUMO+3	−0.62	4.2	71.3	24.5
LUMO+2	−0.65	0.2	99.1	0.7
LUMO+1	−1.16	2.8	96.7	0.5
LUMO	−3.37	74.4	11.5	14.1
HOMO	−5.63	3.2	96.2	0.7
HOMO−1	−6.26	2.6	96.5	0.9
HOMO−2	−7.03	6.9	68.5	24.5
HOMO−3	−7.05	5.8	72.8	21.4

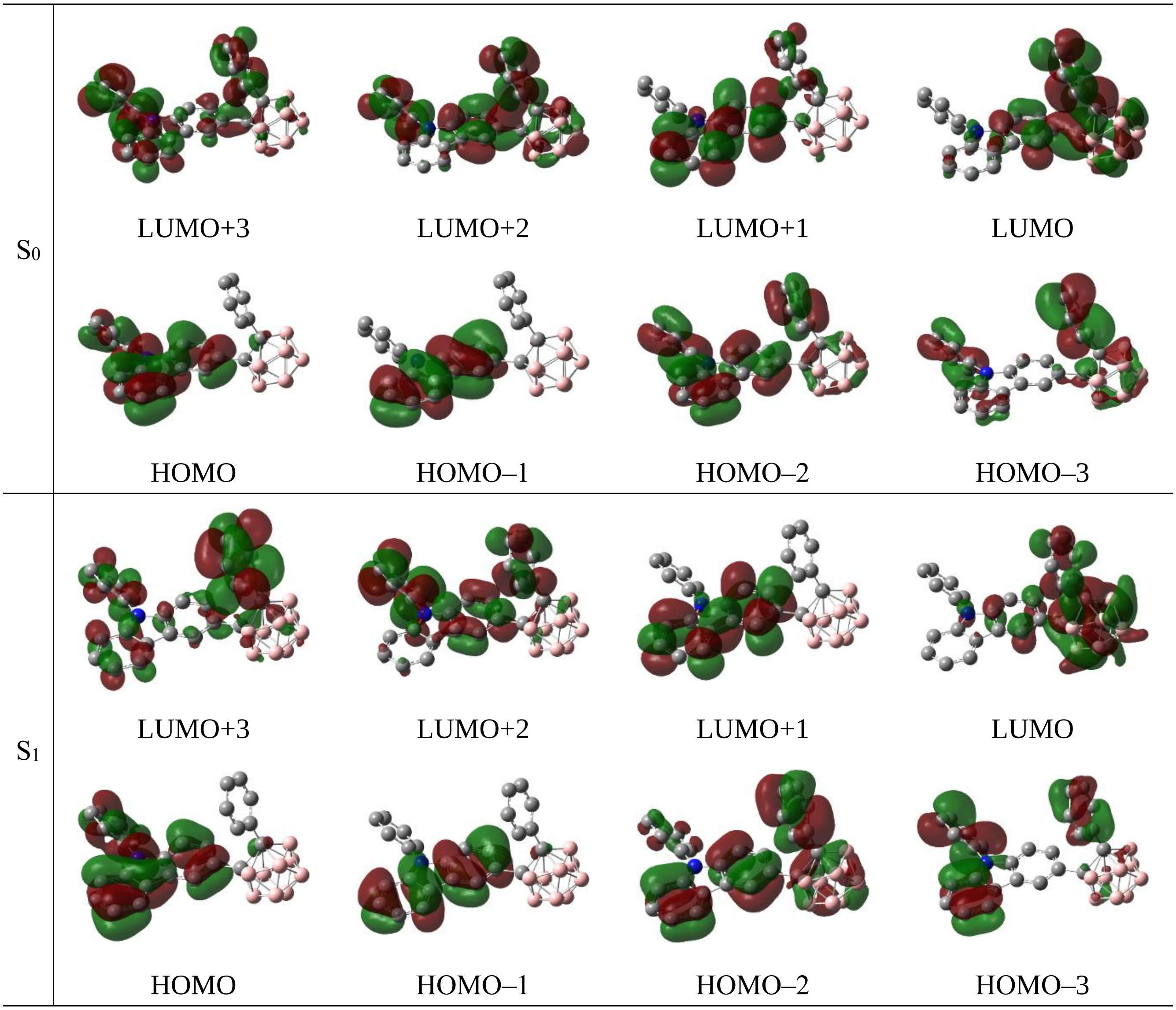

Figure S19. The selected frontier orbitals of **2P** from B3LYP calculations (Isovalue = 0.04 a.u.) at the ground state (S_0) and first singlet excited state (S_1) optimized geometries in THF.

Table S5. Computed absorption wavelengths (λ_{calc} in nm) and oscillator strengths ($f_{calc.}$) for **2P** from TD-B3LYP calculations using the B3LYP geometries at the ground state (S_0) and first singlet excited state (S_1) optimized geometries in THF

state	λ_{calc} (/nm)	f_{calc}	Major contribution
			S_0
1	322.91	0.1173	HOMO → LUMO (87.5%)
			HOMO → LUMO+1 (8.4%)
2	313.88	0.0780	HOMO−1 → LUMO (9.1%)
			HOMO → LUMO (8.9%)
			HOMO → LUMO+1 (76.5%)
3	290.48	0.0271	HOMO−1 → LUMO (79.5%)
			HOMO → LUMO+1 (8.8%)
4	280.78	0.2283	HOMO−1 → LUMO (7.5%)
			HOMO−1 → LUMO+1 (15.9%)
			HOMO → LUMO+2 (70.8%)
5	276.23	0.1613	HOMO−1 → LUMO+1 (37.2%)
			HOMO → LUMO+3 (46.4%)
			S_1
1	559.81	0.2930	HOMO → LUMO (98.7%)
2	415.38	0.0709	HOMO−1 → LUMO (98.3%)
3	388.99	0.1331	HOMO−2 → LUMO (95.3%)
4	381.86	0.0056	HOMO−3 → LUMO (96.5%)
5	365.50	0.1934	HOMO−6 → LUMO (18.1%)
			HOMO−4 → LUMO (78.1%)

Table S6. Molecular orbital energies (in eV) and molecular orbital distributions (in %) of **2P** at the ground state (S_0) and first singlet excited state (S_1) optimized geometries in THF

	E (eV)	*o*-carborane	phenyl carbazole	carborane phenyl
		S_0		
LUMO+3	−0.48	5.5	78.1	16.4
LUMO+2	−0.69	12.8	61.1	26.0
LUMO+1	−1.13	5.0	88.8	6.2
LUMO	−1.36	34.2	41.2	24.6
HOMO	−5.73	2.6	97.4	0.1
HOMO−1	−6.14	1.2	98.6	0.2
HOMO−2	−7.09	4.6	77.2	18.2
HOMO−3	−7.14	9.1	19.4	71.5
		S_1		
LUMO+3	−0.48	6.2	83.6	10.2
LUMO+2	−0.64	3.1	41.2	55.8
LUMO+1	−1.13	2.8	96.7	0.5
LUMO	−3.36	74.4	25.2	0.4
HOMO	−5.62	0.1	93.1	6.8
HOMO−1	−6.23	2.8	96.1	1.1
HOMO−2	−7.03	10.9	85.6	3.5
HOMO−3	−7.15	3.6	38.5	57.9

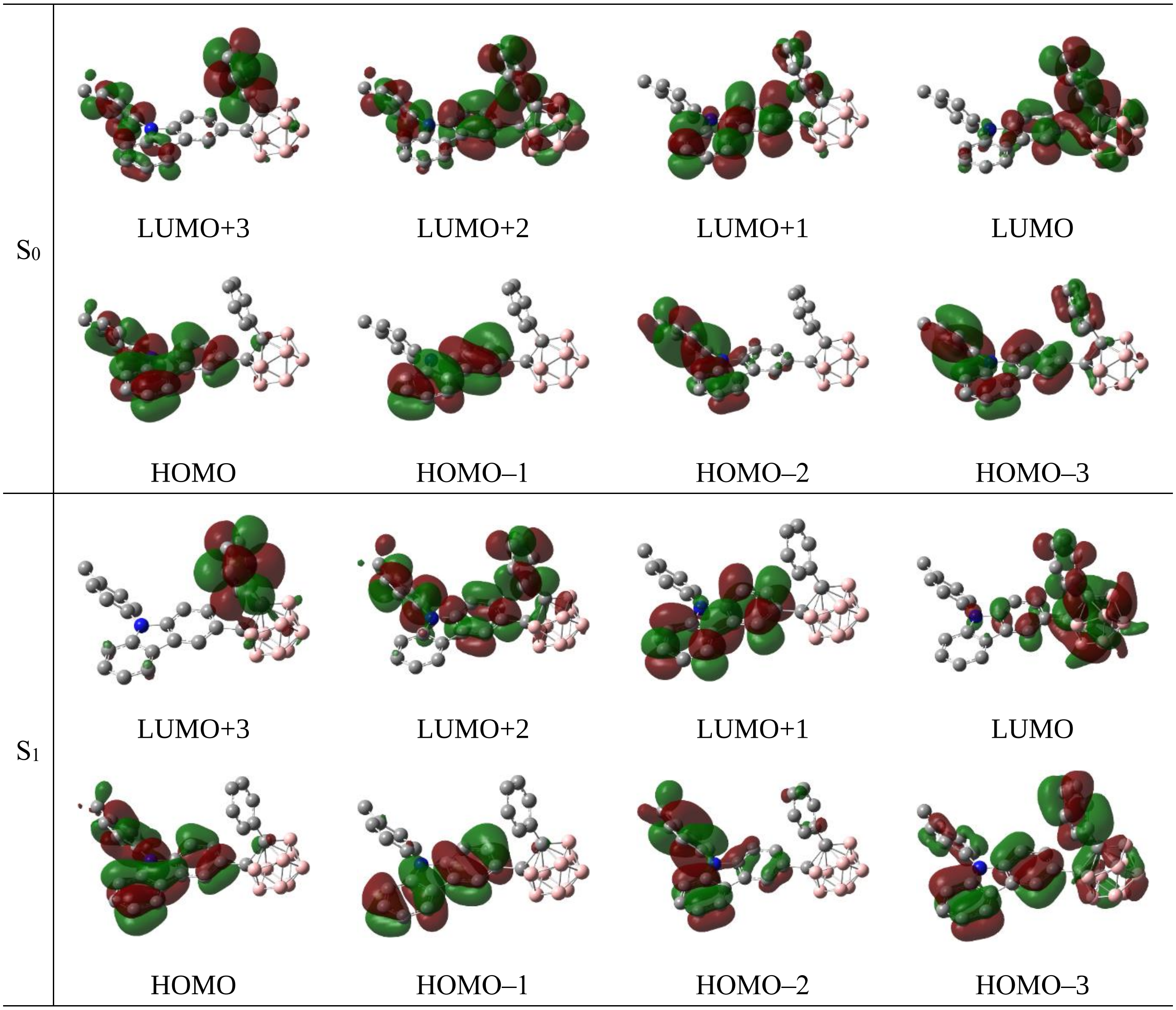

Figure S20. The selected frontier orbitals of **3M** from B3LYP calculations (Isovalue = 0.04 a.u.) at the ground state (S_0) and first singlet excited state (S_1) optimized geometries in THF.

Table S7. Computed absorption wavelengths (λ_{calc} in nm) and oscillator strengths ($f_{calc.}$) for **3M** from TD-B3LYP calculations using the B3LYP geometries at the ground state (S_0) and first singlet excited state (S_1) optimized geometries in THF

state	λ_{calc} (/nm)	f_{calc}	Major contribution
		S_0	
1	325.93	0.1327	HOMO → LUMO (89.3%)
			HOMO → LUMO+1 (6.9%)
2	314.75	0.0793	HOMO−1 → LUMO (9.1%)
			HOMO → LUMO (7.4%)
			HOMO → LUMO+1 (78.2%)
3	291.84	0.1179	HOMO−1 → LUMO (80.3%)
			HOMO → LUMO+1 (8.9%)
4	280.73	0.0978	HOMO−1 → LUMO (7.9%)
			HOMO−1 → LUMO+1 (26.1%)
			HOMO → LUMO+2 (61.2%)
5	273.99	0.1599	HOMO−1 → LUMO+1 (27.0%)
			HOMO → LUMO+2 (12.1%)
			HOMO → LUMO+3 (10.8%)
			HOMO → LUMO+4 (44.1%)
		S_1	
1	571.87	0.2157	HOMO → LUMO (98.9%)
2	416.76	0.0484	HOMO−1 → LUMO (98.4%)
3	391.75	0.1081	HOMO−3 → LUMO (38.0%)
			HOMO−2 → LUMO (59.2%)
4	385.31	0.0763	HOMO−3 → LUMO (57.9%)
			HOMO−2 → LUMO (40.3%)
5	381.15	0.0048	HOMO−4 → LUMO (96.8%)

Table S8. Molecular orbital energies (in eV) and molecular orbital distributions (in %) of **3M** at the ground state (S_0) and first singlet excited state (S_1) optimized geometries in THF

	E (eV)	*o*-carborane	phenyl carbazole	carborane phenyl
		S_0		
LUMO+3	−0.44	5.7	23.9	70.5
LUMO+2	−0.66	15.9	54.7	29.4
LUMO+1	−1.10	5.6	87.6	6.9
LUMO	−1.36	33.7	41.7	24.5
HOMO	−5.69	2.5	97.4	0.1
HOMO−1	−6.12	1.2	98.6	0.3
HOMO−2	−6.93	0.2	99.7	0.1
HOMO−3	−7.07	3.1	90.0	6.9
		S_1		
LUMO+3	−0.48	6.6	2.3	91.0
LUMO+2	−0.60	4.7	67.1	28.2
LUMO+1	−1.13	2.8	96.7	0.6
LUMO	−3.35	74.3	11.6	14.1
HOMO	−5.56	3.3	96.1	0.7
HOMO−1	−6.22	2.4	96.8	0.8
HOMO−2	−6.90	1.3	96.0	2.7
HOMO−3	−7.02	10.3	53.5	36.2

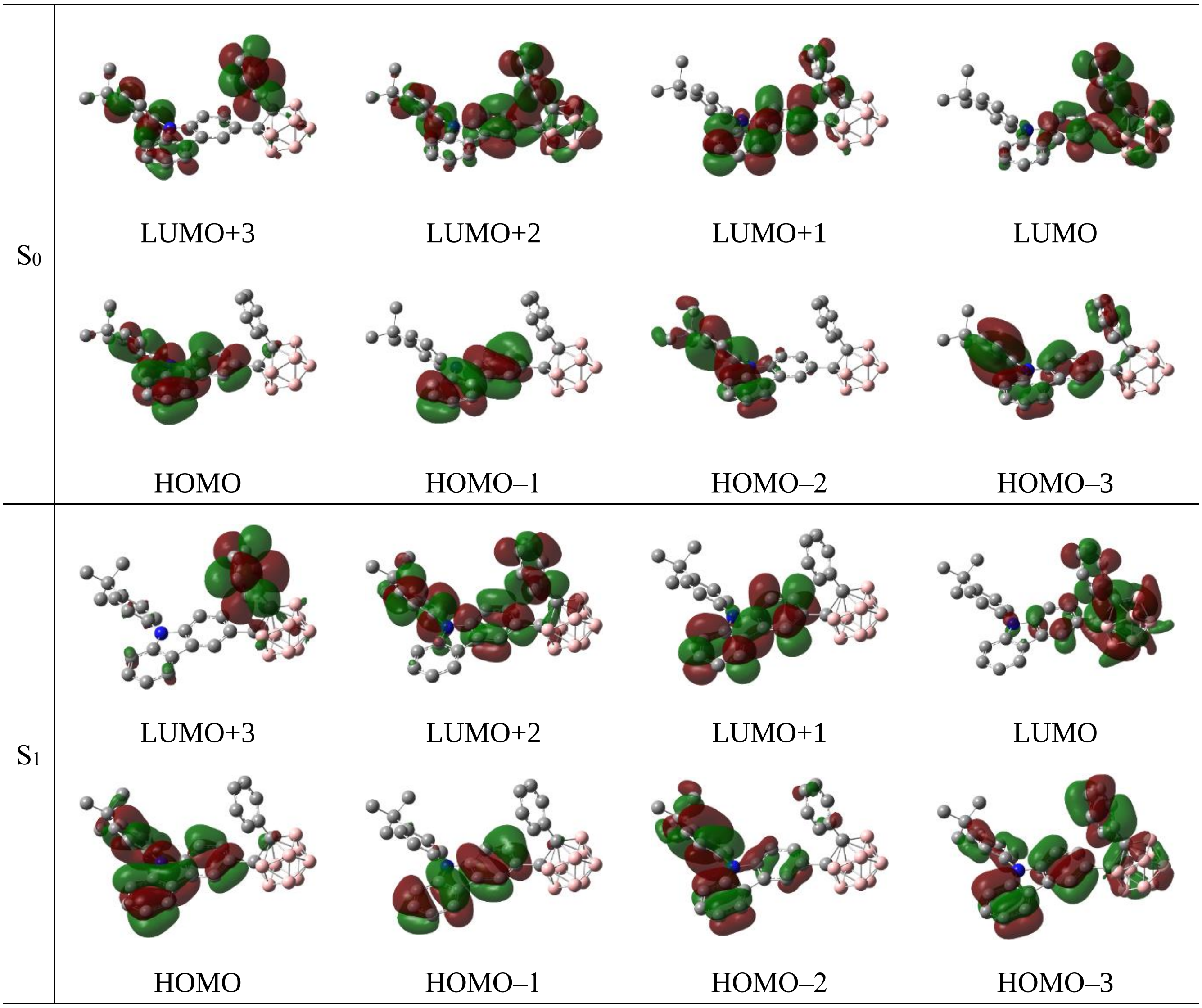

Figure S21. The selected frontier orbitals of **4T** from B3LYP calculations (Isovalue = 0.04 a.u.) at the ground state (S_0) and first singlet excited state (S_1) optimized geometries in THF.

Table S9. Computed absorption wavelengths (λ_{calc} in nm) and oscillator strengths ($f_{calc.}$) for **4T** from TD-B3LYP calculations using the B3LYP geometries at the ground state (S_0) and first singlet excited state (S_1) optimized geometries in THF

state	λ_{calc} (/nm)	f_{calc}	Major contribution		
			S_0		
1	325.79	0.1368	HOMO	→	LUMO (89.1%)
			HOMO	→	LUMO+1 (7.1%)
2	315.08	0.0843	HOMO−1	→	LUMO (8.6%)
			HOMO	→	LUMO (7.5%)
			HOMO	→	LUMO+1 (78.5%)
3	291.67	0.1138	HOMO−1	→	LUMO (80.5%)
			HOMO	→	LUMO+1 (8.4%)
4	280.93	0.0152	HOMO−1	→	LUMO (8.0%)
			HOMO−1	→	LUMO+1 (24.4%)
			HOMO	→	LUMO+2 (62.9%)
5	275.00	0.2435	HOMO−1	→	LUMO+1 (26.8%)
			HOMO	→	LUMO+2 (10.4%)
			HOMO	→	LUMO+3 (20.8%)
			HOMO	→	LUMO+4 (36.8%)
			S_1		
1	570.79	0.2188	HOMO	→	LUMO (98.9%)
2	416.28	0.1510	HOMO−1	→	LUMO (98.4%)
3	392.01	0.0711	HOMO−3	→	LUMO (33.3%)
			HOMO−2	→	LUMO (63.3%)
4	384.88	0.0818	HOMO−3	→	LUMO (61.4%)
			HOMO−2	→	LUMO (36.1%)
5	380.89	0.0045	HOMO−4	→	LUMO (96.9%)

Table S10. Molecular orbital energies (in eV) and molecular orbital distributions (in %) of **4T** at the ground state (S_0) and first singlet excited state (S_1) optimized geometries in THF

	E (eV)	*o*-carborane	phenyl carbazole	carborane phenyl
		S_0		
LUMO+3	−0.44	5.1	37.0	57.9
LUMO+2	−0.66	15.4	55.6	29.0
LUMO+1	−1.11	5.4	87.8	6.8
LUMO	−1.36	33.9	41.4	24.7
HOMO	−5.69	2.5	97.5	0.1
HOMO−1	−6.12	1.2	98.6	0.2
HOMO−2	−6.92	0.2	99.8	0.1
HOMO−3	−7.06	2.5	92.2	5.3
		S_1		
LUMO+3	−0.48	6.6	4.0	89.5
LUMO+2	−0.60	4.5	68.7	26.8
LUMO+1	−1.13	2.8	96.6	0.6
LUMO	−3.35	74.3	11.6	14.1
HOMO	−5.57	3.2	96.0	0.7
HOMO−1	−6.22	2.4	96.7	0.8
HOMO−2	−6.90	1.4	95.7	2.9
HOMO−3	−7.02	9.5	57.2	33.3

Table S11. Cartesian coordinates of the ground state (S_0) fully optimized geometry of **1F** in THF from B3LYP calculations (in Å)

Atom	X	Y	Z
C	-2.329663	-1.719487	3.609734
C	-2.168139	-0.549105	4.352001
C	-2.822642	-1.664000	2.307051
C	-2.512976	0.677859	3.784181
C	-3.159374	-0.433532	1.721228
C	-3.006287	0.736405	2.481723
C	-3.732984	-0.376295	0.329894
C	-2.633733	-0.189742	-1.064202
C	-0.349374	-1.257724	-0.858489
C	-1.152475	-0.093389	-0.833534
C	1.028839	-1.207411	-0.692832
C	4.813238	-0.667021	0.906566
C	-0.540757	1.153387	-0.649417
C	5.884715	-1.557189	0.951155
C	1.622282	0.043481	-0.502709
C	0.841562	1.228863	-0.478214
C	4.046331	-0.546799	-0.258795
C	1.757504	2.328821	-0.254349
C	3.053089	1.762117	-0.149321
C	6.157735	-2.329184	-0.172543
C	1.598749	3.715877	-0.143273
C	4.187563	2.552798	0.050933
C	2.724010	4.508623	0.064231
C	4.002551	3.929744	0.157341
C	4.348749	-1.327811	-1.380804
C	5.405897	-2.235368	-1.338185
H	-2.075623	-2.681678	4.043184
H	-1.783669	-0.593475	5.366265
H	-5.516542	-2.051106	0.692369
H	-2.953487	-2.584922	1.752788
H	-2.402445	1.594893	4.354279
H	-2.795492	-2.602821	-0.367312
H	-5.188103	-2.791024	-2.272311
H	-3.281184	1.696960	2.064348
H	-5.745312	0.939325	0.910102
H	-7.095412	-0.500565	-1.502281
H	-0.811551	-2.223011	-1.020767
H	4.566399	-0.068296	1.776618
H	1.618784	-2.116469	-0.715233
H	6.493356	-1.665423	1.841725
H	-2.517812	-1.541985	-3.132367
H	-1.130410	2.061733	-0.645677
H	-3.153178	2.044918	-0.029124
H	-5.212137	-0.181401	-3.902395
H	-5.566974	2.139924	-1.912441
H	0.613548	4.166186	-0.221085
H	5.176381	2.113169	0.119342
H	2.616399	5.585071	0.152704
H	4.867007	4.567877	0.313833
H	-2.746910	1.447908	-2.914826
H	3.761047	-1.219405	-2.285968
H	5.656481	-2.851967	-2.193928
B	-5.061731	-1.335944	-0.132664
B	-3.429515	-1.687310	-0.744525
B	-4.814945	-1.750772	-1.840509
B	-5.196598	0.427617	-0.004256
B	-5.916353	-0.418099	-1.392155
B	-3.288048	-1.034678	-2.391848
B	-3.643507	1.107083	-0.541532
B	-4.826961	-0.233200	-2.780725
B	-5.035255	1.117027	-1.631770
B	-3.423788	0.728639	-2.264166
N	2.957571	0.372701	-0.302347
F	7.188990	-3.199849	-0.130100

Table S12. Cartesian coordinates of the first excited state (S_1) fully optimized geometry of **1F** in THF from B3LYP calculations (in Å)

Atom	X	Y	Z
C	-3.387663	-3.220360	2.665225
C	-3.334003	-2.509289	3.867801
C	-3.624828	-2.558284	1.463724
C	-3.526675	-1.124496	3.852170
C	-3.820304	-1.160832	1.429901
C	-3.764439	-0.458554	2.653076
C	-4.086449	-0.462331	0.166056
C	-2.383274	0.441611	-1.248645
C	-0.145015	-0.704455	-1.388535
C	-0.914497	0.387984	-0.965005
C	1.235994	-0.791471	-1.146317
C	5.067118	-0.916618	0.725376
C	-0.275603	1.440278	-0.273875
C	6.090359	-1.821616	0.474358
C	1.834616	0.266826	-0.478647
C	1.091135	1.371619	-0.025293
C	4.233667	-0.493570	-0.324651
C	2.018574	2.240066	0.683174
C	3.303772	1.609977	0.624076
C	6.264449	-2.292833	-0.825176
C	1.886658	3.460708	1.328770
C	4.456154	2.216251	1.169849
C	3.025611	4.047039	1.898275
C	4.293373	3.432274	1.810973
C	4.425342	-0.985726	-1.627564
C	5.448617	-1.890036	-1.880595
H	-3.245237	-4.297349	2.663545
H	-3.147387	-3.026984	4.803794
H	-5.785903	-1.898031	-0.753746
H	-3.667136	-3.122632	0.538474
H	-3.493354	-0.560416	4.780029
H	-2.781475	-2.018092	-1.342099
H	-4.935854	-1.299331	-3.487489
H	-3.916205	0.615409	2.655742
H	-5.961696	0.894127	0.829883
H	-6.889891	0.641221	-2.089171
H	-0.627575	-1.519676	-1.913699
H	4.886972	-0.567351	1.735281
H	1.791746	-1.665877	-1.462306
H	6.736358	-2.176722	1.268538
H	-2.261222	-0.024090	-3.626164
H	-0.853685	2.290341	0.068950
H	-3.010667	1.738058	0.787285
H	-4.770694	1.772054	-3.851946
H	-5.202389	3.010054	-1.046542
H	0.925388	3.958738	1.389425
H	5.432237	1.756712	1.078353
H	2.934558	5.001364	2.405352
H	5.155415	3.927412	2.243429
H	-2.431316	2.726564	-2.049902
H	3.801105	-0.631161	-2.439113
H	5.628746	-2.272493	-2.878307
B	-5.145401	-0.922151	-0.977468
B	-3.332087	-0.966318	-1.338325
B	-4.591315	-0.566192	-2.614547
B	-5.244893	0.648819	-0.085837
B	-5.737789	0.549183	-1.799375
B	-3.016046	0.221493	-2.741297
B	-3.466688	1.148778	-0.137429
B	-4.485481	1.214610	-2.838446
B	-4.746978	1.921101	-1.204762
B	-3.114350	1.772478	-1.855654
N	3.193413	0.429541	-0.070767
F	7.251586	-3.168069	-1.068617

Table S13. Cartesian coordinates of the ground state (S_0) fully optimized geometry of **2P** in THF from B3LYP calculations (in Å)

Atom	X	Y	Z
C	-2.204536	-1.707724	3.589076
C	-1.963756	-0.562070	4.348234
C	-2.694645	-1.599829	2.288563
C	-2.226288	0.693652	3.799549
C	-2.948682	-0.340934	1.721860
C	-2.716284	0.804619	2.499290
C	-3.519086	-0.223491	0.333059
C	-2.411431	-0.100536	-1.062497
C	-0.213417	-1.340156	-0.883042
C	-0.926645	-0.119301	-0.836127
C	1.165192	-1.396486	-0.723739
C	5.021821	-1.130266	0.849306
C	-0.221879	1.074506	-0.635063
C	6.020407	-2.104208	0.867184
C	1.852986	-0.197080	-0.518211
C	1.162898	1.042666	-0.470019
C	4.226648	-0.972738	-0.291846
C	2.159012	2.066998	-0.231925
C	3.408916	1.403320	-0.140855
C	6.216080	-2.932708	-0.240027
C	2.103621	3.460157	-0.099570
C	4.599551	2.105794	0.064377
C	3.285032	4.164418	0.114228
C	4.517391	3.490849	0.191952
C	4.426641	-1.794010	-1.407475
C	5.414887	-2.778252	-1.373351
H	-2.014934	-2.691138	4.007585
H	-1.581561	-0.647286	5.360747
H	-5.421863	-1.765213	0.678234
H	-2.887893	-2.501450	1.721008
H	-2.053601	1.592569	4.382912
H	-2.751783	-2.504000	-0.397643
H	-5.156003	-2.486854	-2.297275
H	-2.926625	1.787573	2.096774
H	-5.426297	1.230788	0.936638
H	-6.885008	-0.070677	-1.490514
H	-0.747465	-2.265281	-1.058048
H	4.849545	-0.498565	1.714418
H	1.684317	-2.347058	-0.762860
H	6.637122	-2.222287	1.752750
H	-2.401759	-1.429360	-3.148734
H	-0.741188	2.024606	-0.612887
H	-2.760404	2.151974	0.005329
H	-4.988207	0.139439	-3.891891
H	-5.164175	2.453102	-1.869459
H	1.153992	3.982879	-0.166757
H	5.553605	1.593881	0.120539
H	3.257842	5.244424	0.219145
H	5.427552	4.060698	0.352942
H	-2.405900	1.566351	-2.889962
H	3.817085	-1.652975	-2.293927
H	5.565511	-3.415644	-2.239098
B	-4.916624	-1.074764	-0.138493
B	-3.316454	-1.538454	-0.760028
B	-4.705249	-1.483379	-1.852639
B	-4.919081	0.691944	0.013931
B	-5.702850	-0.078365	-1.382936
B	-3.130231	-0.875991	-2.399053
B	-3.320455	1.260748	-0.518725
B	-4.605532	0.043606	-2.772251
B	-4.709701	1.389593	-1.604373
B	-3.133113	0.890705	-2.246888
N	3.209823	0.027513	-0.317206
H	6.989176	-3.694454	-0.219847

Table S14. Cartesian coordinates of the first excited state (S_1) fully optimized geometry of **2P** in THF from B3LYP calculations (in Å)

Atom	X	Y	Z
C	3.310945	3.951263	1.160183
C	3.181064	3.813572	2.545414
C	3.528740	2.835018	0.357235
C	3.277477	2.541504	3.117741
C	3.627721	1.543733	0.918490
C	3.495583	1.422703	2.318623
C	3.873070	0.366863	0.075457
C	2.142742	-0.950733	-0.918616
C	-0.000903	0.177720	-1.608590
C	0.675145	-0.704226	-0.755184
C	-1.378118	0.433484	-1.490629
C	-5.218331	1.522217	-0.016721
C	-0.056909	-1.363657	0.256516
C	-6.163356	2.315214	-0.661076
C	-2.068543	-0.240631	-0.495219
C	-1.420776	-1.123650	0.387779
C	-4.409805	0.661909	-0.776133
C	-2.429029	-1.595109	1.323399
C	-3.662578	-0.961099	0.956432
C	-6.303567	2.256972	-2.050595
C	-2.408180	-2.477334	2.393386
C	-4.872498	-1.237931	1.632189
C	-3.603548	-2.728411	3.081049
C	-4.819139	-2.118708	2.697284
C	-4.542409	0.601513	-2.172364
C	-5.492670	1.400506	-2.801237
H	3.243485	4.934344	0.702791
H	3.009704	4.685002	3.170041
H	5.691083	1.189768	-1.271327
H	3.630858	2.956636	-0.715679
H	3.184094	2.420761	4.193348
H	2.718485	1.218330	-2.007784
H	4.862986	-0.450237	-3.547332
H	3.572305	0.442219	2.776026
H	5.631773	-0.688127	1.336908
H	6.641401	-1.731305	-1.371649
H	0.552834	0.692360	-2.384251
H	-5.077200	1.591402	1.055951
H	-1.862077	1.142309	-2.151309
H	-6.780777	2.990115	-0.077932
H	2.107540	-1.517137	-3.277179
H	0.447917	-2.044162	0.932194
H	2.628375	-1.308789	1.500565
H	4.488131	-3.377585	-2.608582
H	4.767416	-3.350046	0.475125
H	-1.489991	-2.970990	2.691680
H	-5.806459	-0.788315	1.319541
H	-3.599449	-3.416824	3.919021
H	-5.726664	-2.355353	3.241013
H	2.047074	-3.360387	-0.693574
H	-3.933950	-0.088617	-2.745347
H	-5.606900	1.345875	-3.878585
B	4.987819	0.246874	-1.100853
B	3.191127	0.235796	-1.538250
B	4.447139	-0.730400	-2.467107
B	4.955058	-0.809454	0.367435
B	5.492368	-1.463269	-1.204869
B	2.822932	-1.411040	-2.333740
B	3.147289	-1.186808	0.439286
B	4.220345	-2.432361	-1.934715
B	4.393778	-2.403991	-0.144291
B	2.791329	-2.451499	-0.879440
N	-3.445727	-0.152257	-0.128921
H	-7.040622	2.879100	-2.547368

Table S15. Cartesian coordinates of the ground state (S_0) fully optimized geometry of **3M** in THF from B3LYP calculations (in Å)

Atom	X	Y	Z
C	-2.301202	-0.936489	3.860550
C	-2.219936	0.363331	4.361059
C	-2.781198	-1.165888	2.571986
C	-2.631503	1.433123	3.565372
C	-3.184460	-0.095828	1.758042
C	-3.111170	1.206861	2.276353
C	-3.743963	-0.339957	0.381349
C	-2.633049	-0.419523	-1.015298
C	-0.334935	-1.400704	-0.636308
C	-1.155419	-0.259427	-0.799385
C	1.041785	-1.304244	-0.481398
C	4.843600	-0.449262	0.980141
C	-0.562807	1.009725	-0.816221
C	5.927771	-1.309765	1.144204
C	1.617041	-0.030229	-0.494560
C	0.817894	1.131927	-0.658678
C	4.049884	-0.540688	-0.167985
C	1.716864	2.266774	-0.612902
C	3.020537	1.742763	-0.419350
C	6.232936	-2.287884	0.187251
C	1.537177	3.651220	-0.724904
C	4.142610	2.573028	-0.349677
C	2.649935	4.483902	-0.647852
C	3.936856	3.946231	-0.464722
C	4.346840	-1.501308	-1.140348
C	5.422797	-2.367724	-0.953980
H	-1.994214	-1.778580	4.472805
H	-1.845636	0.540481	5.364569
H	-5.503962	-1.937431	1.065221
H	-2.849001	-2.183239	2.207847
H	-2.583516	2.448461	3.946126
H	-2.761232	-2.643397	0.155612
H	-5.132593	-3.258356	-1.685590
H	-3.438119	2.049149	1.679374
H	-5.783281	1.030130	0.666600
H	-7.085015	-0.892737	-1.410312
H	-0.783095	-2.386163	-0.641838
H	4.608581	0.290272	1.738709
H	1.645780	-2.195773	-0.358546
H	6.541227	-1.224998	2.037055
H	-2.475994	-2.162427	-2.763751
H	-1.165568	1.898280	-0.957015
H	-3.200795	1.968303	-0.457203
H	-5.182862	-1.036103	-3.812116
H	-5.595681	1.635211	-2.338006
H	0.545349	4.068049	-0.873254
H	5.137591	2.164642	-0.212690
H	2.526146	5.558951	-0.732452
H	4.791536	4.614034	-0.413204
H	-2.754009	0.805128	-3.161055
H	3.744122	-1.558930	-2.040960
H	5.644199	-3.109367	-1.716594
B	-5.052809	-1.397150	0.114582
B	-3.408526	-1.835108	-0.401361
B	-4.781224	-2.145449	-1.471390
B	-5.216140	0.352854	-0.119880
B	-5.908512	-0.769315	-1.311320
B	-3.260699	-1.528422	-2.146437
B	-3.668604	0.937058	-0.773972
B	-4.808234	-0.851153	-2.700930
B	-5.049579	0.700443	-1.852352
B	-3.426055	0.221294	-2.382305
N	2.946338	0.345863	-0.349186
C	7.384758	-3.242477	0.391498
H	8.187386	-2.781253	0.973863
H	7.060932	-4.138155	0.935760
H	7.801418	-3.575433	-0.563262

Table S16. Cartesian coordinates of the first excited state (S_1) fully optimized geometry of **3M** in THF from B3LYP calculations (in Å)

Atom	X	Y	Z
C	-3.369924	-3.217924	2.689061
C	-3.320320	-2.497435	3.886209
C	-3.612987	-2.566677	1.482858
C	-3.522977	-1.114217	3.860337
C	-3.818706	-1.170950	1.438728
C	-3.766687	-0.459055	2.656526
C	-4.091531	-0.484065	0.170045
C	-2.396689	0.421692	-1.251494
C	-0.150389	-0.712539	-1.373342
C	-0.927129	0.381983	-0.966825
C	1.230388	-0.786468	-1.130376
C	5.084742	-0.830713	0.738390
C	-0.295605	1.448368	-0.292653
C	6.110291	-1.733963	0.493701
C	1.823634	0.287046	-0.481207
C	1.072006	1.393110	-0.044232
C	4.225147	-0.455749	-0.308927
C	1.992920	2.278944	0.651651
C	3.281082	1.658388	0.604331
C	6.310308	-2.285567	-0.783179
C	1.849999	3.510172	1.275407
C	4.427211	2.285254	1.136901
C	2.982888	4.116884	1.835212
C	4.254403	3.511899	1.757575
C	4.406997	-0.997866	-1.593473
C	5.441993	-1.895465	-1.817486
H	-3.219598	-4.293827	2.695343
H	-3.128986	-3.006684	4.825879
H	-5.780161	-1.940153	-0.737396
H	-3.651926	-3.138246	0.561915
H	-3.492688	-0.542906	4.783864
H	-2.776786	-2.042007	-1.327226
H	-4.936721	-1.355550	-3.476444
H	-3.925984	0.613795	2.651258
H	-5.976982	0.862404	0.825122
H	-6.905153	0.580169	-2.091540
H	-0.627970	-1.539278	-1.884927
H	4.918421	-0.447618	1.738649
H	1.791609	-1.662568	-1.431116
H	6.759975	-2.029987	1.311603
H	-2.271854	-0.061353	-3.625454
H	-0.879185	2.299274	0.038712
H	-3.033261	1.729734	0.774316
H	-4.795238	1.714224	-3.864026
H	-5.235713	2.970049	-1.068197
H	0.884178	4.000788	1.325444
H	5.407787	1.834488	1.050901
H	2.883154	5.079329	2.325195
H	5.113256	4.021448	2.179793
H	-2.462923	2.700077	-2.070044
H	3.771957	-0.678136	-2.411458
H	5.589701	-2.292170	-2.817190
B	-5.147624	-0.960928	-0.969099
B	-3.334635	-0.994137	-1.330967
B	-4.597579	-0.613114	-2.609247
B	-5.258925	0.615933	-0.089317
B	-5.752155	0.499398	-1.801874
B	-3.028138	0.185486	-2.742203
B	-3.485217	1.130024	-0.145580
B	-4.505534	1.166581	-2.846448
B	-4.771987	1.883453	-1.218269
B	-3.138284	1.742180	-1.868296
N	3.181070	0.463879	-0.073860
C	7.405549	-3.288190	-1.030346
H	8.242933	-3.143114	-0.343111
H	7.032210	-4.308986	-0.880910
H	7.779176	-3.226118	-2.055787

Table S17. Cartesian coordinates of the ground state (S_0) fully optimized geometry of **4T** in THF from B3LYP calculations (in Å)

Atom	X	Y	Z
C	-2.815745	-1.397137	3.778764
C	-2.880794	-0.140120	4.380810
C	-3.288613	-1.580815	2.480351
C	-3.431865	0.931236	3.677018
C	-3.831882	-0.506649	1.758236
C	-3.905452	0.750562	2.378503
C	-4.383246	-0.711174	0.371857
C	-3.306487	-0.508953	-1.038615
C	-0.880396	-1.164782	-0.768725
C	-1.863897	-0.149117	-0.826273
C	0.470259	-0.877222	-0.621208
C	4.138704	0.422334	0.871561
C	-1.465609	1.191153	-0.741897
C	5.337632	-0.278998	0.957419
C	0.850880	0.465015	-0.532812
C	-0.114913	1.504330	-0.588762
C	3.339051	0.299805	-0.270397
C	0.606740	2.753006	-0.455172
C	1.977747	2.415690	-0.321085
C	5.773411	-1.134021	-0.070919
C	0.220968	4.099305	-0.445758
C	2.965687	3.396025	-0.193650
C	1.199463	5.079946	-0.310112
C	2.555899	4.727697	-0.188534
C	3.753676	-0.535441	-1.308944
C	4.950290	-1.246899	-1.200096
H	-2.399355	-2.241531	4.318981
H	-2.511706	0.001739	5.391831
H	-5.872753	-2.603321	0.935945
H	-3.241308	-2.567175	2.036248
H	-3.498297	1.912000	4.137308
H	-3.078447	-2.816089	-0.060625
H	-5.377524	-3.621200	-1.921922
H	-4.342098	1.591441	1.854267
H	-6.593901	0.312623	0.797870
H	-7.648923	-1.602899	-1.421994
H	-1.178205	-2.202233	-0.852491
H	3.816092	1.059100	1.688960
H	1.201769	-1.675936	-0.580661
H	5.937542	-0.162133	1.854418
H	-2.937246	-2.057160	-2.932657
H	-2.195663	1.988797	-0.801026
H	-4.205672	1.714066	-0.272761
H	-5.804447	-1.261349	-3.849022
H	-6.569422	1.186786	-2.146389
H	-0.825139	4.373424	-0.546296
H	4.013258	3.131459	-0.103681
H	0.915664	6.127603	-0.300737
H	3.303124	5.509389	-0.090034
H	-3.657499	0.859014	-3.069438
H	3.146958	-0.623296	-2.204502
H	5.238447	-1.887597	-2.024811
B	-5.528413	-1.924805	0.030324
B	-3.850467	-2.070008	-0.540305
B	-5.188202	-2.490223	-1.617055
B	-5.953096	-0.205137	-0.050805
B	-6.501314	-1.314780	-1.326325
B	-3.791513	-1.600465	-2.254049
B	-4.524349	0.654248	-0.669829
B	-5.434494	-1.116324	-2.730375
B	-5.880832	0.306030	-1.748920
B	-4.217464	0.118874	-2.336265
N	2.113006	1.022387	-0.370078
C	7.103018	-1.895852	0.075239
C	7.410629	-2.775138	-1.151271
H	8.362304	-3.293928	-0.999744
H	6.641150	-3.537553	-1.310410
H	7.500527	-2.181636	-2.066908
C	8.259252	-0.881803	0.246153
H	8.123266	-0.252908	1.130860
H	9.212044	-1.410880	0.356963
H	8.335678	-0.223967	-0.625833
C	7.035521	-2.810297	1.321785
H	6.229355	-3.545002	1.225582
H	7.978497	-3.354686	1.442129
H	6.862046	-2.238829	2.238342

Table S18. Cartesian coordinates of the first excited state (S_1) fully optimized geometry of **4T** in THF from B3LYP calculations (in Å)

Atom	X	Y	Z
C	-3.717225	-2.889562	3.229844
C	-3.830910	-1.957791	4.266009
C	-3.998735	-2.522842	1.916700
C	-4.236150	-0.652706	3.970044
C	-4.407844	-1.209491	1.600321
C	-4.518913	-0.281788	2.658461
C	-4.720466	-0.824340	0.218379
C	-3.114124	0.045586	-1.322406
C	-0.723397	-0.736144	-1.188281
C	-1.669231	0.283773	-1.010085
C	0.640583	-0.553828	-0.910570
C	4.366398	0.325965	1.003635
C	-1.231553	1.539658	-0.539044
C	5.522632	-0.445398	0.957799
C	1.040325	0.698257	-0.465410
C	0.116651	1.739460	-0.260254
C	3.513052	0.371420	-0.109148
C	0.866218	2.867672	0.271519
C	2.231451	2.450401	0.367286
C	5.870747	-1.189037	-0.182610
C	0.518345	4.157469	0.646746
C	3.249053	3.329887	0.793123
C	1.524587	5.020717	1.102241
C	2.872244	4.609997	1.165841
C	3.834549	-0.367976	-1.261173
C	4.995580	-1.125093	-1.286620
H	-3.408629	-3.908453	3.446265
H	-3.609372	-2.245051	5.289397
H	-6.133710	-2.666577	-0.418373
H	-3.909367	-3.256674	1.123073
H	-4.334105	0.079923	4.766207
H	-3.123846	-2.417114	-0.927105
H	-5.260693	-2.486987	-3.204645
H	-4.835843	0.732965	2.443416
H	-6.811410	0.316576	0.568811
H	-7.553070	-0.649384	-2.253139
H	-1.051429	-1.705701	-1.542722
H	4.099628	0.853392	1.912447
H	1.336915	-1.374682	-1.029857
H	6.149196	-0.474439	1.840221
H	-2.810385	-0.852016	-3.555476
H	-1.947749	2.338174	-0.384869
H	-4.029331	1.599849	0.400917
H	-5.553264	0.438745	-4.176070
H	-6.302610	2.117125	-1.683925
H	-0.509185	4.498516	0.583826
H	4.287171	3.023023	0.813472
H	1.264277	6.031290	1.397778
H	3.627778	5.312096	1.500089
H	-3.476375	2.090979	-2.567983
H	3.200681	-0.309430	-2.138337
H	5.230625	-1.669080	-2.194866
B	-5.641779	-1.663713	-0.824506
B	-3.829207	-1.486977	-1.142674
B	-5.074352	-1.551440	-2.491655
B	-6.023734	0.014962	-0.268527
B	-6.415239	-0.496714	-1.933959
B	-3.634587	-0.561631	-2.749606
B	-4.344796	0.775322	-0.393230
B	-5.233608	0.144046	-3.066862
B	-5.677428	1.105998	-1.613259
B	-4.012286	1.096572	-2.196044
N	2.336997	1.150060	-0.073254
C	7.141174	-2.047500	-0.261688
C	7.958789	-2.004888	1.042605
H	8.850039	-2.629622	0.932749
H	8.295163	-0.990919	1.281576
H	7.389613	-2.390238	1.894700
C	6.746252	-3.518097	-0.542570
H	6.202527	-3.625029	-1.485511
H	7.646304	-4.138353	-0.605686
H	6.115268	-3.915157	0.258981
C	8.033688	-1.525095	-1.414616
H	8.333678	-0.486965	-1.239645
H	8.940939	-2.133797	-1.486587
H	7.524080	-1.571646	-2.381178
